# Exploring physicochemical and cytogenomic diversity of African cowpea and common bean

**DOI:** 10.1038/s41598-021-91929-2

**Published:** 2021-06-18

**Authors:** Sílvia Catarino, Miguel Brilhante, Anyse Pereira Essoh, Alberto B. Charrua, Josefa Rangel, Guilherme Roxo, Eromise Varela, Margarida Moldão, Ana Ribeiro-Barros, Salomão Bandeira, Mónica Moura, Pedro Talhinhas, Maria M. Romeiras

**Affiliations:** 1 Linking Landscape, Environment, Agriculture and Food (LEAF), Instituto Superior de Agronomia (ISA), Universidade de Lisboa, Tapada da Ajuda, 1340-017 Lisbon, Portugal; 2 Forest Research Center (CEF), Instituto Superior de Agronomia (ISA), Universidade de Lisboa, Tapada da Ajuda, 1340-017 Lisbon, Portugal; 3 Centre for Ecology, Evolution and Environmental Changes (cE3c), Faculdade de Ciências, Universidade de Lisboa, Campo Grande, 1749-016 Lisbon, Portugal; 4 Research Centre in Biodiversity and Genetic Resources (CIBIO), InBIO Associate Laboratory, Faculdade de Ciências e Tecnologia, Universidade dos Açores, Ponta Delgada, Portugal; 5 Nova School of Business and Economics, Universidade Nova de Lisboa, Campus de Carcavelos, Rua da Holanda, n.1, Carcavelos, 2775-405 Cascais, Portugal; 6 Department of Earth Sciences and Environment, Faculty of Science and Technology, Licungo University, P.O. Box 2025, 2100 Beira, Mozambique; 7 Centro de Botânica, Universidade Agostinho Neto, Luanda, Angola; 8 Department of Biological Sciences, Eduardo Mondlane University, PO Box 257, 1100 Maputo, Mozambique

**Keywords:** Biochemistry, Ecology, Molecular biology, Plant sciences

## Abstract

In sub-Saharan Africa, grain legumes (pulses) are essential food sources and play an important role in sustainable agriculture. Among the major pulse crops, the native cowpea (*Vigna unguiculata*) and introduced common bean (*Phaseolus vulgaris*) stand out. This paper has two main goals. First, we provide a comprehensive view of the available genetic resources of these genera in Africa, including data on germplasm collections and mapping biodiversity-rich areas. Second, we investigate patterns of physicochemical and cytogenomic variation across Africa to explore the geographical structuring of variation between native and introduced beans. Our results revealed that 73 *Vigna* and 5 *Phaseolus* species occur in tropical regions of Africa, with 8 countries accounting for more than 20 native species. Conversely, germplasm collections are poorly represented when compared to the worldwide collections. Regarding the nuclear DNA content, on average, *V. unguiculata* presents significantly higher values than *P. vulgaris*. Also, *V. unguiculata* is enriched in B, Mg, S, and Zn, while *P. vulgaris* has more Fe, Ca, and Cu. Overall, our study suggests that the physicochemical and cytogenomic diversity of native *Vigna* species is higher than previously thought, representing valuable food resources to reduce food insecurity and hunger, particularly of people living in African developing countries.

## Introduction

Leguminosae (*nom. alt.* Fabaceae family) is characterized by approximately 770 genera and 19,500 species, being largely cultivated worldwide^1,2^. The crops within this family provide an important resource throughout the tropical and temperate world, ranking third among the most economically significant plant families^3^. In sub-Saharan Africa, grain legumes (pulses) are widely cultivated for human consumption due to their considerable content of protein and are occasionally sold for cash income^4^. In addition, dry beans are important sources of energy (starch), dietary fiber and minerals. Moreover, through the consociation of cultures they are used as a cheaper means to enrich the soil with nitrogen (N), one of the main limiting factors in crop production worldwide, particularly in this region as a result of the intensive cultivation observed over the past two centuries^5,6^.

The most widely consumed legume crops in this sub-region are *Arachis hypogaea* L. (groundnut), *Cajanus cajan* (L.) Huth (pigeon pea), *Cicer arietinum* L. (chickpea), *Phaseolus vulgaris* L. (common bean), and *Vigna unguiculata* (L.) Walp. (cowpea)^7^. The common bean and cowpea are the most cultivated pulses, constituting important staples and food security resources due to their potential to alleviate malnutrition^8^.

The genus *Phaseolus*, originates from the tropics and subtropics of the New World, and includes 89 accepted species^9^. In many Latin American and African countries, common bean (*P. vulgaris*) is the most economically important source of protein^10^. It has its center of origin in the Mesoamerican region^11^ and was probably first introduced in eastern Africa by the Portuguese in the sixteenth century^12^. Furthermore, Arab slave traders and Swahili merchants carried the common bean inland, which became established as food crop in Africa before the colonial era^13^.

On the other hand, the highest diversity of the genus *Vigna* is found in sub-Saharan Africa, namely in Namibia, Angola, Botswana, Zambia, Zimbabwe, Mozambique, and the Republic of South Africa^14–16^. This genus has 105 accepted species^9^ distributed across tropical regions and includes important legume crops such as *V. radiata* (mung bean) and *V. unguiculata* (cowpea), as well as several neglected and underutilized crops such as *V. aconitifolia* (moth bean) and *V. subterranea* (bambara groundnut)^17^. Hypothetically, Africa is regarded as the center of origin of cultivated cowpea during the Neolithic times, where wild and cultivated relatives can be found displaying a large diversity of forms and remaining the dominant African grain legume^18^. Altogether, its remarkable ability to tolerate abiotic stresses (i.e. drought and high temperatures)^14,19^ and the significant amount of dietary protein (18–35%), vitamins, and minerals it contains, make this kind of beans extremely important both economically and socially across sub-Saharan Africa, which suffers from a chronic protein shortage^20,21^.

Cowpea is usually considered the “poor man’s meat”^22^, as it provides high nutritional value food both for humans and the cattle, and ca. 80% of production occurs in the drier savanna and Sahelian zones of Africa^23,24^. On the other hand, productivity of common bean is extremely low in sub-Saharan Africa, partly due to drought stress, and low levels of phosphorous (P) available in the soil^25^. Soils with low levels of P seems to be related to plants of small genome sizes and therefore, cytogenomic data can be a predictor of soil types or even of farming practices^26^.

Beyond the molecular markers, the use of nuclear DNA content to study the intraspecific genetic diversity and also molecular, cellular, and evolutionary genomic mechanisms has recently been increasing^27^. In this sense, data regarding genome size is of extreme importance for different scientific fields such as systematics and ecology^28^. Estimation of nuclear DNA content is nowadays simple and efficient though the widely used technique, flow cytometry^29^. This technique has been successfully applied to estimate the nuclear DNA size of several plant species such as *P. vulgaris* and *V. unguiculata* (e.g., Parida et al*.*^30^, Nagl and Treviranus^31^; Barow and Meister^32^; Lonardi et al*.*^33^), however, cytogenomic studies targeting intraspecific variability in both species are scarce.

This paper has two main goals. First, we provide a comprehensive view of the available genetic resources of the genera *Vigna* and *Phaseolus* in Africa, including data on germplasm collections (ex situ conservation) and mapping biodiversity-rich areas (in situ conservation), to identify the main diversity hotspots in Africa. Second, we investigate patterns of physicochemical (e.g. seed phenotypic traits and mineral content) and cytogenomic variation across Africa to explore the geographical structuring of variation between native cowpea (*V. unguiculata*) and introduced bean species (*P. vulgaris*). Therefore, samples collected across different African countries (i.e.: Western: Angola and Eastern Africa: Mozambique, and also in the dry islands, located in the Atlantic Ocean ca. 570 km off the western coast of Africa, near Senegal) were analyzed to: (i) characterize seed phenotypic traits (i.e. quantitative: length, width, and height; and qualitative: shape, color and hilum); (ii) determine the profiles of 11 minerals in seeds of these species, and identify homogeneous groups based on their mineral content; and (iii) assess the nuclear DNA content of ecotypes of both species based on flow cytometry and compare the genome size between species and collection sites. In addition, the current and potential contribution of these species as a high valuable food resource in the sub-Saharan Africa region is also discussed.

## Results

### Species diversity in Africa

**Figure 1:**
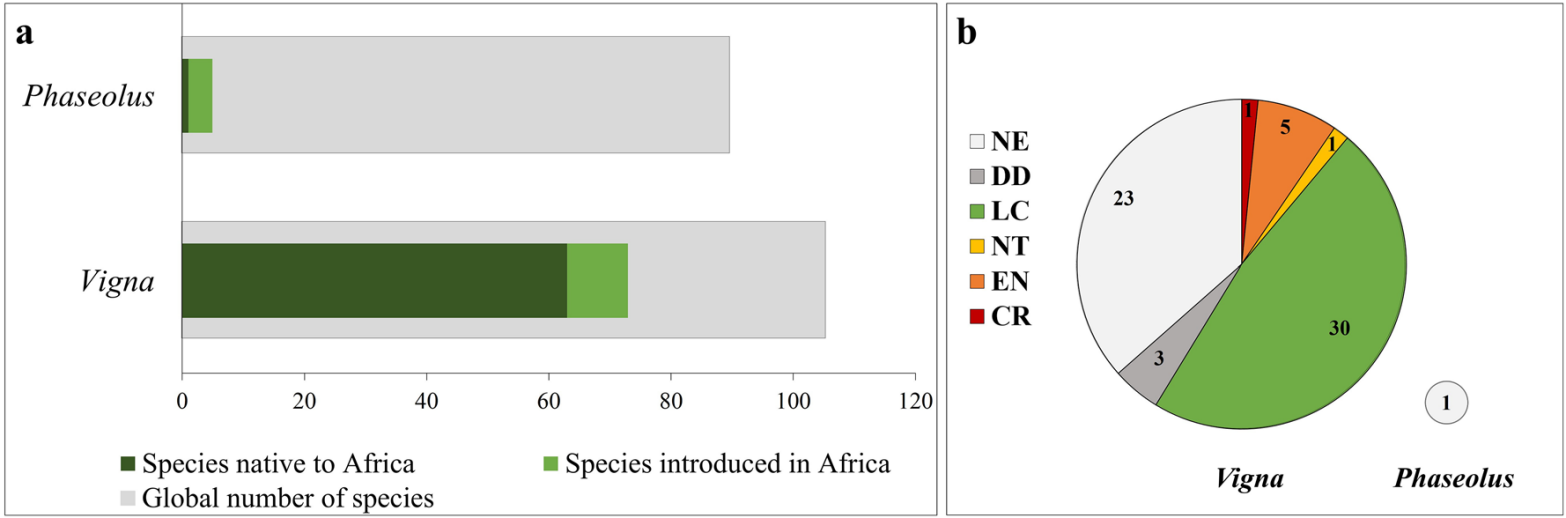
The genera *Vigna* and *Phaseolus* in Africa: (**a**) Number of species of *Vigna* and *Phaseolus* occurring globally (total number of accepted species), occurring in Africa and native to Africa; (**b**) Conservation status of *Vigna* and *Phaseolus* species native to Africa continent (IUCN conservation status: CR, Critically Endangered; EN, Endangered; VU, Vulnerable; NT, Near threatened; LC, Least concern; DD, Data deficient; NE, Not evaluated).

Our study identified 73 *Vigna* species and 5 *Phaseolus* species occurring in Africa (Fig. 1a), representing 69.5% and 5.6% respectively of the total number of species currently accepted. The diversity of *Vigna* is much higher and most species are native to Africa (63 species) (Supplementary Table S1). Thirteen of them are endemic, occurring in only one country, for instance: *V. angivensis*, *V. bosseri*, *V. keraudrenii*, and *V. microsperma* occur in Madagascar; *V*. *mendesii* and *V*. *ramanniana* in Angola; and *V. monantha* and *V. somaliensis* in Somalia. Among native *Vigna*, six are cultivated by African populations (namely, *V. subterranea, V. parkeri, V. unguiculata, V. marina, V. luteola* and *V. vexillata*) mainly for human consumption, but also for animal fodder and medicinal uses. The introduced species of *Vigna* are native from Asia (seven species) and America (3 species), most of them (6 species) being cultivated.

The genus *Phaseolus* has low species diversity in Africa, and four out of the five species identified originate from America. These species are extensively cultivated in Africa mainly for human and animal consumption. Only one species, *P. massaiensis,* is native to Africa (Supplementary Table S1) and is restricted to Tanzania.

### Conservation status and germplasm collections

The information available at the IUCN Red List^34^ revealed that 61.9% of the native species were already evaluated according to the IUCN criteria and categories: one species, *V. dolomitica,* is classified as Critically Endangered, four species as Endangered, one species as Near Threatened, three species as Data Deficient, and 30 species as Least Concern. Twenty-four native species remain unevaluated, including *P. massaiensis* (Fig. 1b).

The analysis of the accessions available worldwide reveals that only 50 of the 78 studied species (64.1%) are preserved in gene banks (Supplementary Table S1). The species *P. vulgaris, V. unguiculata* and the introduced *V. radiata* have the highest number of worldwide accessions, ca. 136,000, 40,000 and 16,000 respectively. Six species (7.7%) have 10,000–1000 accessions, 10 species (12.8%) 1000–100; 15 species (19.2%) 100–10, and 16 species (20.5%) 10–1. The 28 species without accessions have, in general, restricted areas of distribution: 11 are endemic to a single country (i.e., Angola: *V. mendesii* and *V. ramanniana*; Madagascar: *V. bosseri*, *V. keraudrenii* and *V. microsperma*; Tanzania: *P. massaiensis* and *V. jaegeri*; Somalia: *V. monantha* and *V. somaliensis*; Central African Republic: *V. tisserantiana*; and *V. mildbraedii* is endemic to Central Rwanda).

To explore the Angola’s crop diversity conserved in gene banks, we consulted the available data in the Genesys database^35^, which contains 530 accessions of five species, with around 95% of them belonging to *P. vulgaris* (290) and *V. unguiculta* (217). However, most of them are stored at the SADC Plant Genetic Resources Centre in Zambia and none have been reported in Angolan gene banks or in other local Institutions. In Mozambique, 58 accessions were collected (i.e.: 46 of *V. unguiculata*, 8 of *V. radiata* and the remaining belong to three other species). Like Angola, Mozambique does not have accessions registered in national gene banks, and most are stored at the International Institute of Tropical Agriculture in Nigeria. Cabo Verde has only two accessions (*P. lunatus* and *P. vulgaris*) gathered in the country, but both are stored at the Portuguese Plant Germplasm Bank. More details are provided in Supplementary Table S1.

### Biodiversity-rich areas in Africa

**Figure 2:**
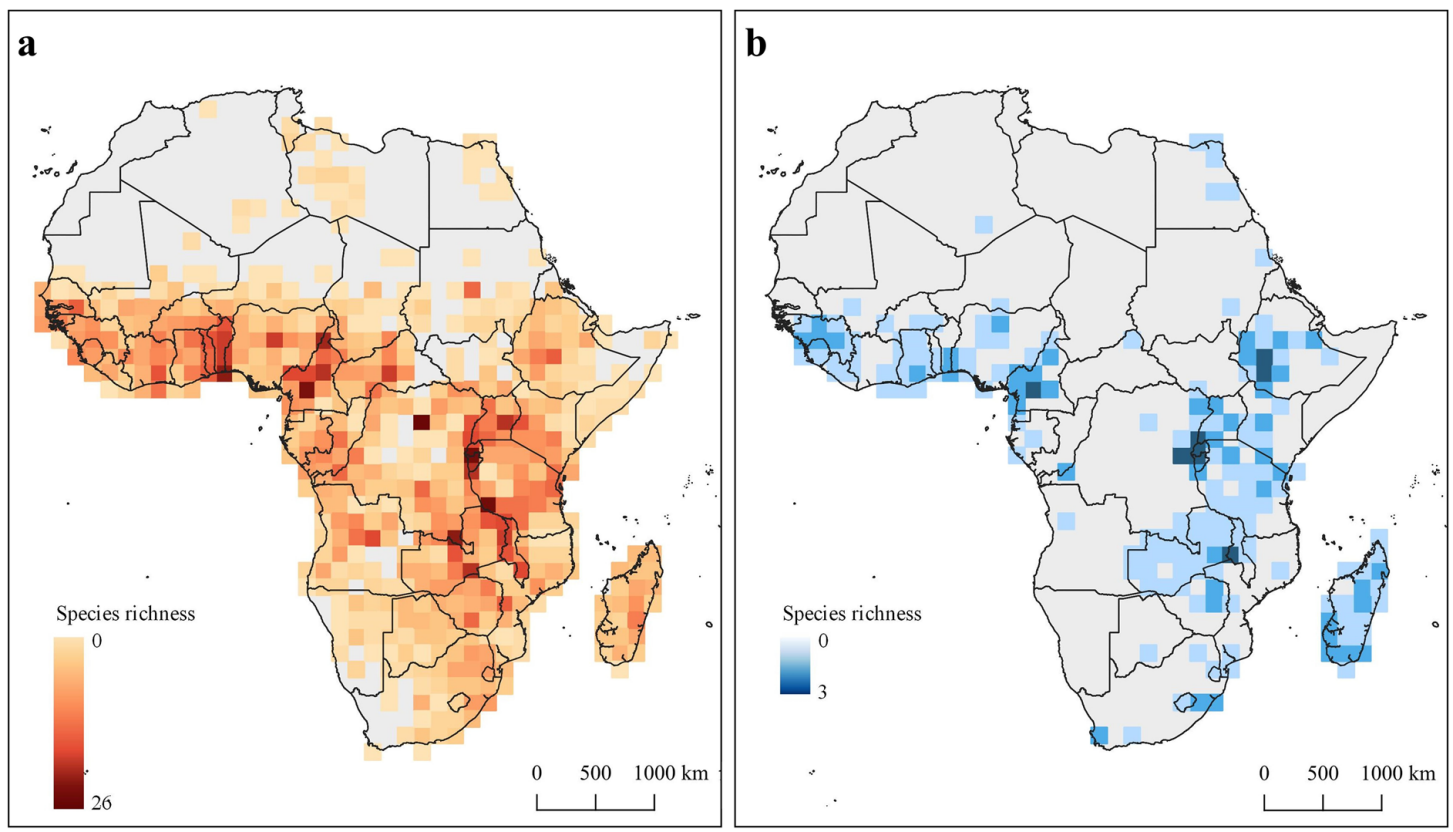
Species richness by cell of 200 × 200 km including native and introduced species of (**a**) *Vigna* and (**b**) *Phaseolus*. This figure was produced with the free available on-line software QGIS v.3.4.4 (http://qgis.osgeo.org).

The highest number of *Vigna* species (native and introduced) were found in Central Africa, with a maximum of 26 species by cell (200 × 200 km) found in the Democratic Republic of Congo (Fig. 2a). Other areas in West and East Africa such as Togo, Benin, Nigeria, Cameroon, Tanzania, Malawi, and Zambia, are also very rich in *Vigna* species. The region of Southern Africa presents low values of richness, with a maximum of 9 species found in a cell. In Africa, the diversity of *Phaseolus* species reaches the maximum of 3 species per cell (Fig. 2b), which is found in Cameroon, Ethiopia, Eastern Democratic Republic of Congo, Rwanda, Burundi, Zambia and Malawi. The number of records recorded in Southern Africa, Namibia and Angola is very low for this genus. The Northern African region presents the lowest species richness for both genera.

**Figure 3:**
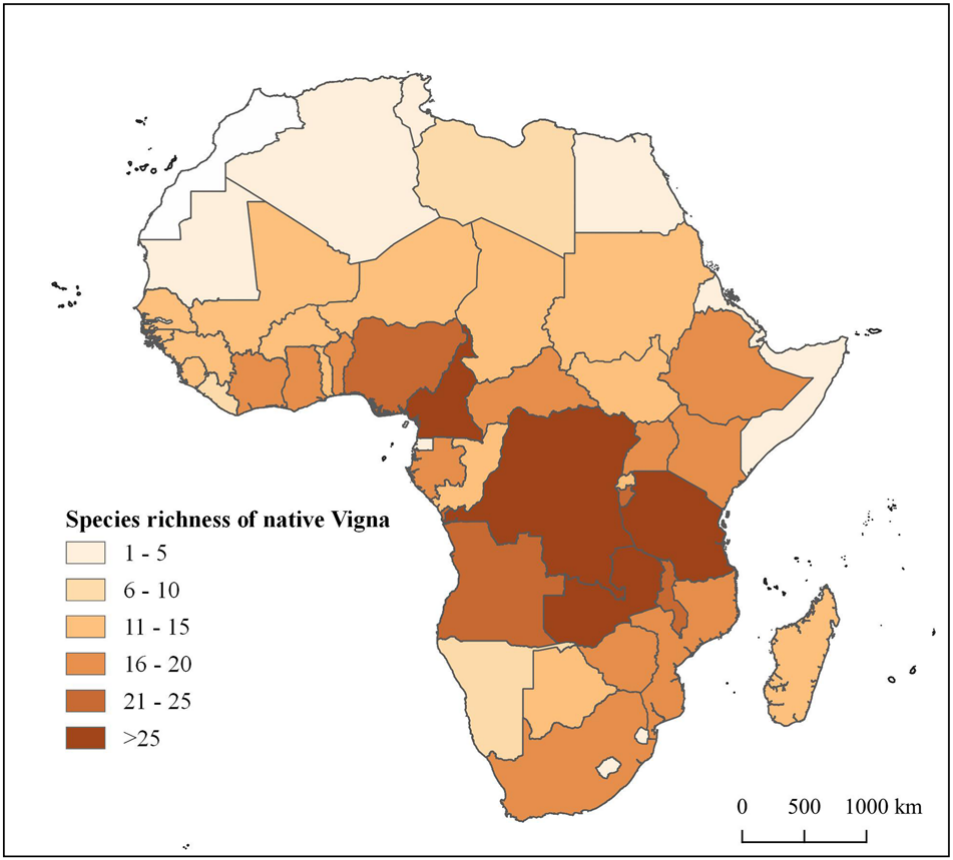
Species richness of native *Vigna* by country. This figure was produced with the free available on-line software QGIS v.3.4.4 (http://qgis.osgeo.org).

In terms of native species, four countries stand out for hosting a high number of *Vigna* species (Fig. 3): Democratic Republic of Congo with 32 species, Tanzania with 30 species, Zambia with 28 species, and Cameroon with 27 species. Burundi, Angola, Nigeria, and Malawi also boast a great diversity with more than 20 native *Vigna* species. These results represent the data available in herbariums and museums worldwide, which is affected by the collection effort in each region over time.

### Physicochemical characterization of *P. vulgaris* and *V. unguiculata*

#### Physical characterization

Our results showed that the studied seeds of *V. unguiculata* and *P. vulgaris* presented a varied array of colors and seed shapes.

*Vigna unguiculata* samples are mostly globose (94%) and most of the samples (82%) presented brown seeds, the hilum was always white, while the color of the rim varied between black and brown, and rarely yellow (MP04Vu). Length values of *V. unguiculata* varied from 5.5 mm (UI31Vu) to 10.3 mm (CC27Vu), width values ranged from 4.7 mm (UI31Vu) to 8.2 mm (MP34Vu) and height values ranged between 3.2 mm (UI31Vu) and 5.9 mm (MP34Vu).

All samples of *P. vulgaris* had an oblong shape. Most of the samples presented brown color (ca. 76%), the hilum was always white and the color of the rim varied between black and brown, and rarely white (SO14Pv). The length of *P. vulgaris* varied from 10.1 mm (MA21Pv) to 17.0 mm (SO11Pv), width values ranged from 6.3 mm (MA43Pv) to 8.5 mm (SO09PV) and height values varied between 4.3 mm (CV38Pv) and 10.1 mm (MP02Pv). Overall, *P. vulgaris* presented higher length, width, and height values (13.5 ± 2.3, 7.1 ± 0.7, and 5.9 ± 3.4 mm, respectively) comparatively to *V. unguiculata* (8.0 ± 1.2, 6.5 ± 1.0, and 4.9 ± 0.8 mm, respectively) (Supplementary Table S3).

#### Mineral content

**Figure 4:**
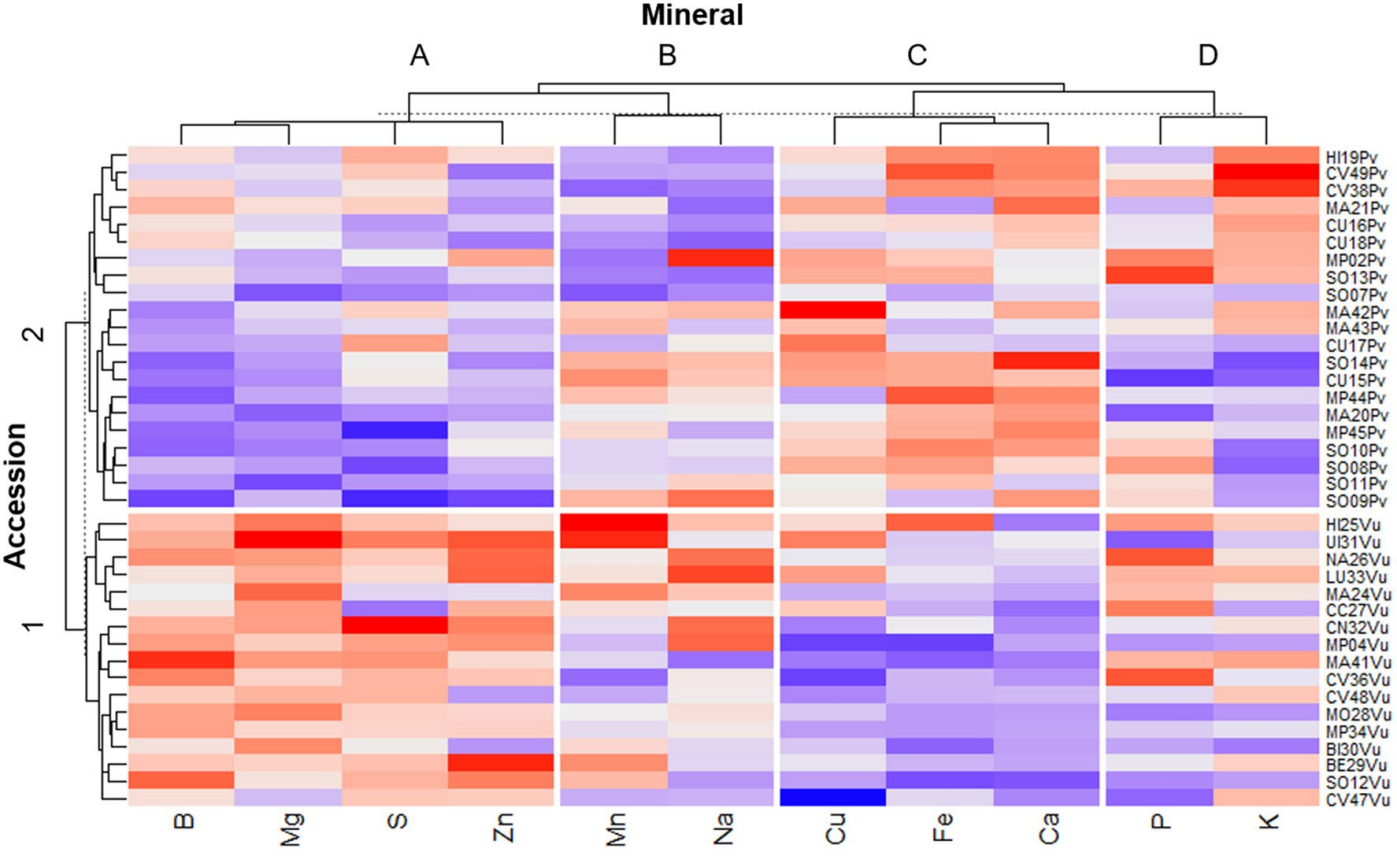
Heatmap of the 21 *Phaseolus vulgaris* and 17 *Vigna unguiculata* accessions obtained from the chemical characterization data for the content of 11 minerals. Red and blue boxes indicate high values and low values respectively.

Mineral diversity was analyzed in 38 samples of *P. vulgaris* (n = 21) and *V. unguiculata* (n = 17) collected in Angola (20), Mozambique (13), and Cabo Verde (5) (Supplementary Table S4). Figure 4 and Supplementary Table S4 represent the average values (mg/kg ww) and homogeneous groups for the content of 11 minerals (Sodium [Na], Potassium [K], Calcium [Ca], Magnesium [Mg], Potassium [P], Sulfur [S], Iron [Fe], Copper [Cu], Zinc [Zn], Manganese [Mn], and Boron [B]) across the 21 *P. vulgaris* and the 17 V*. unguiculata* accessions. The results presented in Supplementary Table S4 reveal that the average Na content ranged between 260.4 (MP02Pv) and 103.0 mg/kg ww (CU18Pv) for *P. vulgaris*, while for *V. unguiculata* the average was ranged from 252.2 (LU33Vu) to 109.6 mg/kg ww (MA41Vu). K content ranged between 11,845.5 (CV49Pv) and 8490.8 mg/kg ww (SO14Pv) for *P. vulgaris*; between 10,656.3 (MA41Vu) and 8865.6 mg/kg ww (BI30Vu) for *V. unguiculata*. The average content of Ca encompassed values from 933.5 (CU17Pv) to 1701.8 mg/kg ww (SO14Pv) in *P. vulgaris* whereas in *V. unguiculata* from 602.7 (SO12Vu) to 1062.2 mg/kg ww (UI31Vu). On average, for *P. vulgaris*, the lowest Mg content was 1492.5 (SO11Pv) and the highest was 1906.4 mg/kg ww (MA21Pv); for *V. unguiculata*, the lowest value was 1746.0 (CV47Vu) and the highest 2340.3 mg/kg ww (UI31Vu). The P content of *P. vulgaris* ranged from 3119.9 (CU15Pv) to 5210.8 mg/kg ww (SO13Pv), while it ranged from 3301.8 (UI31Vu) to 5117.9 mg/kg ww (NA26Vu) for *V. unguiculata*. The S values varied between 1294.7 for accession MP45Pv and 2021.2 mg/kg ww for accession CU17Pv of *P. vulgaris*; for *V. unguiculata* between 1483.1 for accession CC27Vu and 2348.0 mg/kg ww for accession CN32Vu. The Fe contents attained a range of values between 49.0 (MA21Pv) and 79.5 mg/kg ww (CV49Pv) among *P. vulgaris* accessions, and from 39.1 (MP04Vu) to 78.0 mg/kg ww (HI25Vu) in *V. unguiculata*. The Cu values ranged between 6.0 (MP44Pv) and 9.9 mg/kg ww (MA42Pv) in *P. vulgaris* and between 4.1 (CV47Pv) and 8.7 mg/kg ww (UI31Vu) in *V. unguiculata*. Across *P. vulgaris* accessions, the Zn values varied from 17.5 (SO09Pv) to 31.9 mg/kg ww (MP02Pv) whereas in *V. unguiculata* the values ranged from 22.0 (BI30Vu) to 38.5 mg/kg ww (BE29Vu). The *P. vulgaris* accessions showed a Mn content of 13.8 (SO07Pv) to 28.3 mg/kg ww (CU15Pv) while *V. unguiculata* presented values of 14.8 (CV36Vu) to 38.0 mg/kg ww (HI25Vu). Finally, the B content ranged from 0.7 (SO09Pv) to 9.4 mg/kg ww (MA21Pv) for *P. vulgaris* and from 6.8 (MA24Vu) to 14.3 mg/kg ww (MA41Vu) in *V. unguiculata*.

At the species level, *V. unguiculata* accessions presented highest mean values of Na (179.9 mg/kg ww), Mg (2045.0 mg/kg ww), P (4151.9 mg/kg ww), S (1919.6 mg/kg ww), Zn (30.8 mg/kg ww), Mn (23.6 mg/kg ww), and B (9.6 mg/kg ww). Contrariwise, *P. vulgaris* presented the highest values for K (9901.9 mg/kg ww), Ca (1277.8 mg/kg ww), Fe (64.8 mg/kg ww), and Cu (7.5 mg/kg ww).

**Figure 5:**
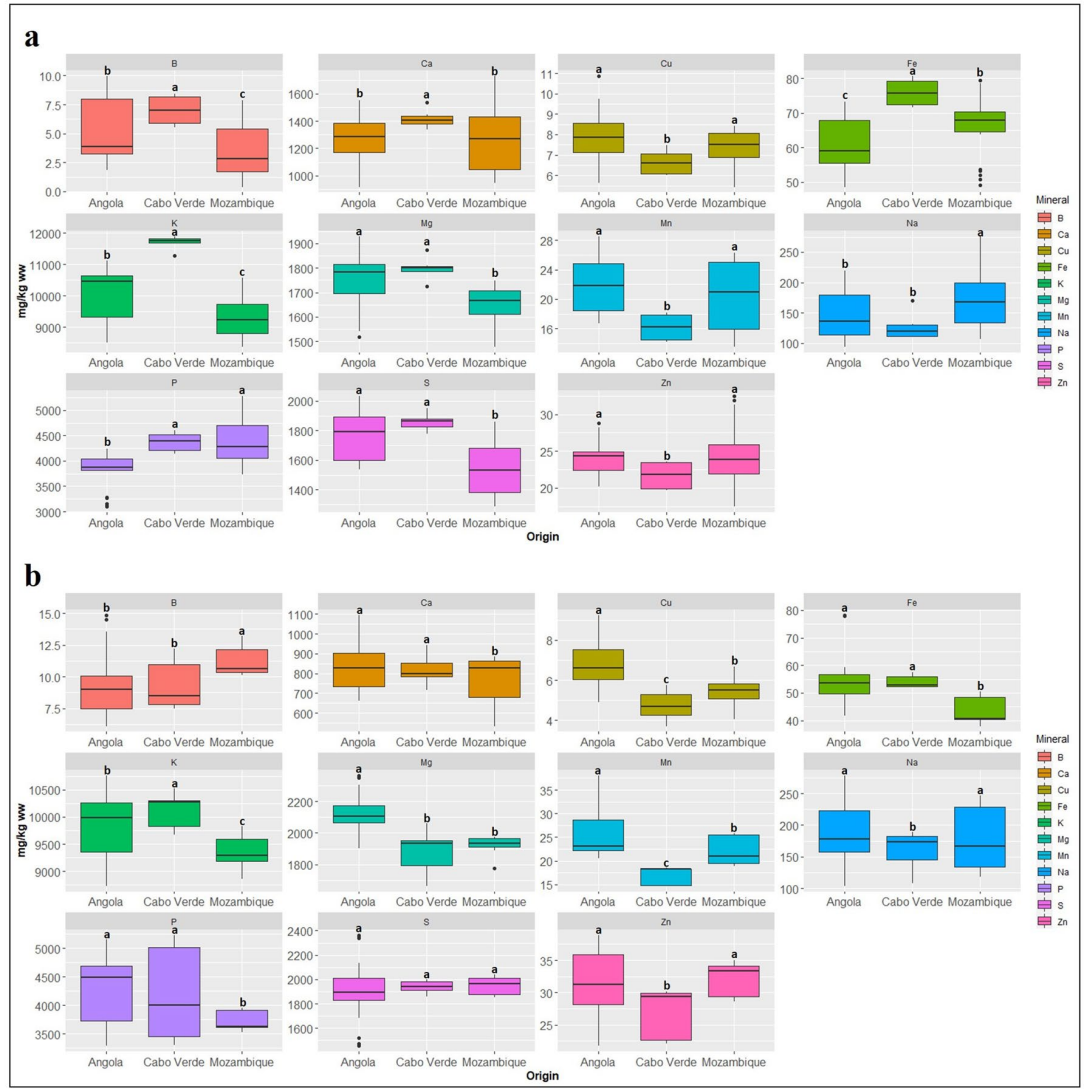
Variation of mineral content (mg/kg ww) and the geographical origin for the studied samples: (**a**) *Phaseolus vulgaris* (21 accessions), and (**b**) *Vigna unguiculata* (17 accessions). Geographical origins sharing the same letter are not statistically different according to the Scott-Knott test at 5% of confidence.

Based on a correlation matrix, a heatmap was constructed (Fig. 4) using Euclidean distances and the UPGMA method, wherein the vertical columns are the clusters of minerals while in the horizontal lines are the clusters of African bean accessions. In the plotted grid, boxes for each factor combination are encoded by dark red colors for the highest values and dark blue for the lowest values of correlation between mineral contents and accessions. The heatmap also plots a dendrogram from a cluster analysis showing the hierarchy of values for both accessions and mineral traits. Four groups of minerals could be discriminated in Fig. 4: Group A encompasses B, Mg, S, and Zn minerals; Group B is composed of Mn and Na minerals; Group C includes Cu, Fe, and Ca; and Group D is defined by P and K. Figure 4 also reveals two clusters of accessions corresponding to the two distinct species: *V. unguiculata* (Cluster 1) and *P. vulgaris* (Cluster 2). Cluster 1 is related to a higher content of B, Mg, S, and Zn minerals defined by cluster A. Cluster 2 is associated with a higher content of Cu, Fe, and Ca minerals defined by cluster C. Cluster 1 represents the 17 V*. unguiculata* accessions which are discriminated by mineral group A. Cluster 2 is composed of 21 *P. vulgaris* accessions defined by group C. Groups B and D did not exhibit a clear pattern for the Clusters 1 and 2. Furthermore, a comparative analysis between the mineral content of the two studied species and the geographical origin of accessions did not reveal a clear pattern (Fig. 5). Nonetheless, Cabo Verde presented extreme values in *P. vulgaris* for the majority of minerals (B, Ca, Cu, Fe, K, Mn, Z) (Fig. 5a), while *V. unguiculata* showed less variation between the geographical origins (Fig. 5b).

### Cytogenomic diversity of *P. vulgaris* and *V. unguiculata*

**Figure 6:**
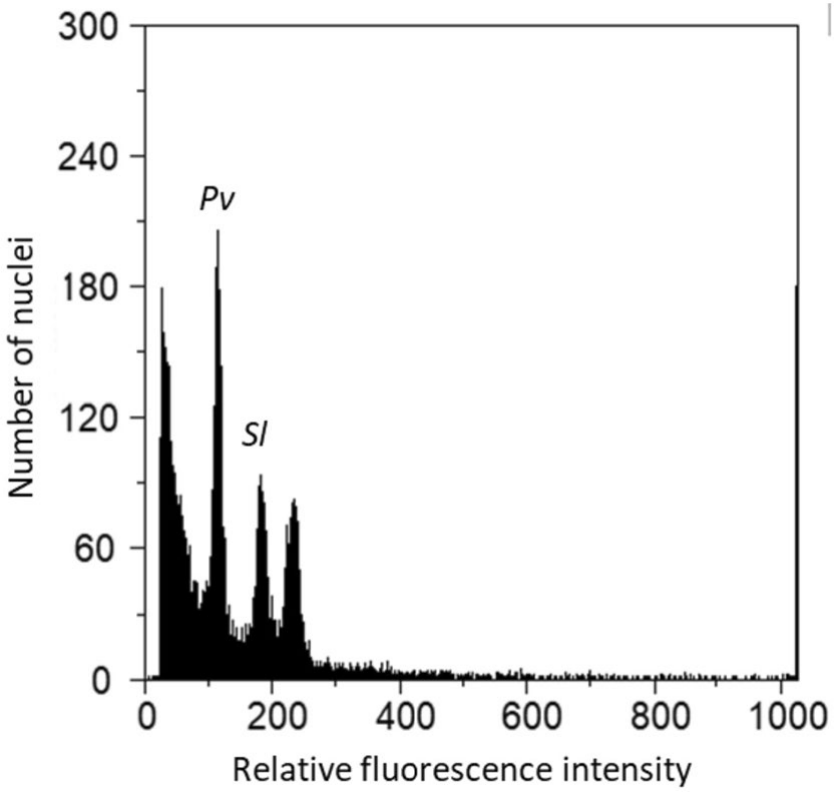
Flow cytometric histogram of relative fluorescence intensities from propidium iodide-stained nuclei of *Phaseolus vulgaris* sample MA20Pv (Pv) and *Solanum lycopersicum* L. (Sl), used as an internal reference standard.

The 38 samples of *P. vulgaris* (n = 21) and *V. unguiculata* (n = 17) collected in Africa were cultivated, 33 of them germinated and their leaves were used for flow cytometric analysis. Figure 6 illustrates a flow histogram presenting the final genome size estimation. Genome size of analyzed samples ranged between 1277.3 Mbp (*V. unguiculata* sample CV47Vu) and 1598.5 Mbp (*V. unguiculata* sample BE29Vu) (Supplementary Table S5). The average genome size of the 13 V*. unguiculata* samples was 1414.7 ± 86.2 Mbp and significantly larger than the average genome size of the 20 *P. vulgaris* samples (1337.4 ± 33.3 Mbp) (Table 1).

**Table 1:** Comparison of the average genome size of the *Phaseolus vulgaris* and *Vigna unguiculata* accessions estimated by flow cytometry.

Species	Genome size (Mbp)		H.G.^b^
	Average	Stdev^a^	
*Vigna unguiculata*	1414.7	86.2	a
*Phaseolus vulgaris*	1337.4	33.3	b

^a^Standard deviation.

^b^Homogeneous groups: accessions sharing the same letter for each mineral are not statistically different according to the Scott-Knott test at 5% of confidence.

There are no significant differences among countries (Angola, Mozambique and Cabo Verde) for *V. unguiculata* and *P. vulgaris* samples (Supplementary Tables S6 and S7). Among *V. unguiculata* samples, those obtained from the Angolan provinces of Bié (1543.4 Mbp) and Benguela (1598.5 Mbp) presented genome sizes larger than the remainder (Supplementary Table S8). The comparison among regions for the *P. vulgaris* accessions evidenced higher average genomes for the Angolan provinces of Cunene (1356.7 Mbp) and Huíla (1342.8 Mbp), and for the Mozambican province of Sofala (1336.4 Mbp) in relation to the remaining provinces under study (Supplementary Table S9).

## Discussion

Our study reveals a much greater diversity of *Vigna* species in Africa (73 species, 63 native) than *Phaseolus* (5 species, 1 native), as expected^14,36^. This great disparity is justified by the original centers of diversity of both genera: *Vigna* has its centers in Africa and Asia^14,37^, while *Phaseolus* has America as its center^38^.

Only one *Phaseolus* species (*P. massaiensis*)^9^ is native to Africa, which is poorly studied and occurs only in Tanzania. The other *Phaseolus* (*P. vulgaris, P. lunatus, P. acutifolius,* and *P. coccineus*) are native from America and grown in Africa, where they were probably introduced during the sixteenth century as a food source^39^.

Based on specimen occurrence data, we found that the areas of the highest diversity of *Vigna* species, located in Western and Eastern Africa, were mainly associated with regions with a tropical climate. Our results are according to previous studies^14,16,40^, which identify these areas as significant biodiversity hotspots of African flora and specifically of *Vigna* genus. Eight countries (i.e. Democratic Republic of the Congo, Tanzania, Zambia, Cameroon, Burundi, Angola, Nigeria, and Malawi) present more than 20 native species, highlighting their central role in the conservation of African *Vigna* diversity.

The conservation status has been accessed over the last few years, but only 60.9% (39 of the 64 native species) were already evaluated according to the IUCN criteria^34^. Among them, six are classified in the threat categories (one critically endangered and five endangered) and one as near threatened, but this number is likely to increase in the near future, because the pressure on natural resources continues to increase in most African countries, driven by economic and population growth as well as climate change^41,42^. Currently, the specific threats to African native pulses species are not well known^14^, but some studies focusing on the conservation of native biodiversity of African legumes^41–43^ identified deforestation, agriculture and urbanization, harvesting for medicinal uses, fires, and invasive species as the main threats. Three species are classified as Data Deficient and 25 remain unevaluated, revealing the lack of knowledge about them. The evaluation of the conservation status, as well as the study of their threats, distribution and ecology, based upon extensive fieldwork, is a required step to protect native species^16,44,45^. Of particular concern are *V. monantha*, *V. bosseri* and *V. keraudreni*, which are classified as endangered and are not preserved in gene banks.

Despite the recognized importance of African native pulses, the information available on ex situ conservation, and the germplasm collections preserved in national institutions of the three studied countries (Angola, Mozambique and Cabo Verde), is still very limited. Nevertheless, it must be highlighted the role that the International Institute of Tropical Agriculture-IITA^46^ have in Africa to keep collections of both crops and non-crops, in (in situ) or out (ex situ) of their natural environment. IITA’s gene bank holds over 28,000 accessions of plant material of major African food crops and maintains the world’s largest assemblage of cowpea germplasm collections with over 15,000 accessions^47^. As stated above, Angola, Mozambique and Cabo Verde revealed a high genetic diversity, however, no accessions of the studied species were reported in the national gene banks. Our results agree with previous studies, which recognized the lack of ex situ conservation strategies for Crop Wild Relatives native to Angola^16^ and Cabo Verde^48,49^. As *Vigna* and *Phaseolus* genera include several cultivated species and their Crop Wild Relatives (i.e., other taxa with a close genetic relationship to cultivated species), their conservation is extremely important to guarantee the supply of new genetic material, essential for the future crop improvement.

The *Vigna* genus includes 13 species cultivated in Africa, which constitute an essential source of nutrients for humans and domestic animals. Most of these species (7) are native to Asia and have only recently been introduced to Africa^14^. However, the cultivation of *Vigna* in Africa mainly focuses on two native species, *V. unguiculata* and *V. subterranea*.

*Vigna unguiculata* is one of the most important sub-Saharan Africa indigenous grain legumes, having been firstly domesticated in Northeast Africa, and secondarily in West Africa and in the Indian sub-continent^15,50^. Cowpea matures earlier than cereals, becoming an important source of income for the rural population before maize, millets and cassava are harvested^14,24^. It is widely cultivated in almost all African countries and was probably introduced around 300 BC in Europe, 200 BC in India, and in the seventeenth century to tropical America by the Spanish^9^.

Mineral content analyses revealed that *V. unguiculata* and *P. vulgaris* form an excellent source of minerals. *Vigna unguiculata* had, on average, higher content of B, Mg, S, and Zn, while *P. vulgaris* had more Fe, Ca, and Cu. Fe content in *P. vulgaris* ranged from 49.0 mg/kg (MA21Pv) to 79.5 mg/kg (CV49Pv) and 39.1 mg/kg (MP04Vu) to 78.0 mg/kg (HI25Vu) in *V. unguiculata*. For *P. vulgaris* these values were lower than the Fe contents reported by Di Bella et al*.*^51^ and Gelin et al*.*^52^, which were on average 86.2 mg/kg and 86.9 mg/kg, respectively; whilst for *V. unguiculata* the contents tallied with Dakora and Belane’s study^53^ where the obtained values ranged from 61 mg/kg to 67 mg/kg. Moreover, Yeken et al*.*^54^ found much higher values of Cu, Zn, Mn, Fe in biofortified *P. vulgaris* but lower values of Ca and Mg, for which our accessions show higher content. However, Gondwe et al*.*^55^ focusing on *V. unguiculata* from Swaziland, found much lower contents of Ca, Fe, and Zn than the content found in our study, even for improved varieties. Content variation within the same species can be explained by several cross-related reasons: (i) the nutritional content of legumes can vary greatly and is highly dependent on soil fertility, which directly influences the supply and availability of most nutrient elements^56^; (ii) the nutritional content is dependent and vary with different varieties and genotypes^46,57^; and (iii) the nutritional content is influenced by preharvest conditions of the plant, maturity of the edible product at harvest and postharvest handling and storage conditions^58^. Additionally, three different aspects account for the content of micronutrients in the seeds, namely micronutrient uptake, transport and remobilization from other parts of the plant, and storage capacity^56^. The mechanisms and proteins involved in all these processes differ for each species due to different evolutionary processes.

Our results highlight the importance of *P. vulgaris* and *V. unguiculata* as sources of essential micronutrients. Chronic deficiency of essential vitamins and minerals is a scourge affecting more than two billion people globally and account for approximately 7% of the global disease burden^59^. This form of undernutrition is known as the ‘hidden hunger’ and occurs due an insufficient intake and absorption of essential vitamins and minerals^60^. Deficiencies in zinc, iron, and iodine have the strongest negative impact on public health, but other minerals such as calcium, selenium, magnesium, and fluoride, also contribute to the health burden^61^. Research carried out by Muthayya et al*.*^62^ which calculated the Hidden Hunger Index (HHI-PD) for preschool-age children revealed that sub-Saharan Africa was the most affected region with high rates of anemia due to iron and vitamin-A deficiency. Moreover, a study by Joy et al.^63^ identified the top three mineral deficiencies, Ca being the highest, followed by Zn and I (Iodine). Regarding Fe deficiency, recent studies have noted an increasing percentage over the last 5 years, from 24.8%^64^ to 54%^65^. Therefore, the inclusion of grain beans in their diets will aid the fight against food insecurity and other several forms of malnutrition.

Analyzing the mean values of the results based on the nutrient reference values for daily reference intakes for minerals (Adults) [NRVs] values reported by the European Union^66^, it appears that *V. unguiculata* and *P. vulgaris* are an excellent mineral source. A usual intake of 100 mg per day is enough to reach about 50% of NRVs of K, *V. unguiculata* (49.0%) and *P. vulgaris* (49.5%); more than 50% of P, *V. unguiculata* (59.3%) and *P. vulgaris* (58.9%) and for Mg, *V. unguiculata* (54.5%) and *P. vulgaris* (45.5%).

Regarding cytogenomic diversity, our study reveals a higher genome size variation in *V. unguiculata* (1414.7 ± 86.2 Mbp) when compared with *P. vulgaris* (1337.4 ± 33.3 Mbp). However, our results do not reveal a correlation between the genome size variation and the studied locations (i.e. Eastern region of Africa: Mozambique; Western region of Africa: Angola, and in the Northeastern Atlantic Ocean: Cabo Verde Islands). Particularly, very small intraspecific variation in nuclear DNA content was found among *P. vulgaris* samples collected from different regions and different habitats (e.g. samples collected in humid zones of Malanje, and others in semi-arid zones of Cunene, close to the Kalahari Desert; see Supplementary Table S2). This species is introduced and more domesticated than *V. unguiculata,* a pattern observed in other crop species, like *Daucus* species as demonstrated by Nowicka et al*.*^67^. Guilengue et al*.*^68^ found a 9.2% genome size variability among a tarwi (*Lupinus mutabilis* Sweet) collection, an Andean crop that is believed to have been domesticated ca. 2600 years ago. The genome size variability of the *P. vulgaris* accessions analyzed in the present study is of 7.8%, suggesting that *P. vulgaris* might have undergone selective pressures within the studied African countries associated with the domestication process. On the other hand, *V. unguiculata* accessions analyzed in this study present a 17.6% genome size variability, showing a higher diversity and illustrating the native nature of this crop in Africa.

Crop genetic diversity is highly dynamic. Differences could be related to several factors such as agroecological conditions of cultivation, seed selection and management by the preferences of farmers in seed selection and the management of seed lots, among others, which have important effects on the chemical composition of the grains and population genetics^69^. Moreover, climate and growth conditions may act as mutagenic factors, causing structural changes in chromosomes (addicting and deleting regions), which result in differences in DNA among different samples of the same species^70^.

Comparative molecular cytogenetic analysis of native and introduced lineages could provide important information on possible karyotypic reorganization and evolution based upon regional environmental stresses. Modern plant-breeding and crop improvement programmes must be conducted following a holistic methodology and research focusing on only one aspect of a crop will not provide enough information to decide on which breeding technology may be more suitable. An initial step of plant genome mapping is the accurate estimation of nuclear genome sizes^71,72^. Nutrient content and genome size are known to be connected to life form, with geophytes being among the extreme examples of large genomes, linked, among other factors, to nutrient storage capacity, while genome size is usually reduced in annual species such as therophytes^73^. The life strategy of a species and a particular environment may limit genome size increase such as in improved polyploids, or the genome size may cause a species to adopt specific life strategies or colonize certain environments^73^. A priori information on distribution, genome size, capacity of storage of mineral content are thus fundamental data which allow breeding companies and governmental agricultural stakeholders to determine the applicability and interest of implementing an improvement or simple breeding programme.

## Final remarks

Based on extensive research, our study exposes the great diversity of native and introduced *Vigna* and *Phaseolus* species in Africa. Our results highlight the importance of *V. unguiculata* and *P. vulgaris* as sources of essential nutrients, making them a vital resource for the poorest populations. If complemented with further investigation on genetic traits, this study may also be relevant to increase the efficiency of breeding programs for both commercial production and smallholder farming systems in Africa.

Thus, the device of holistic strategies (e.g., combining socio-economy; ecogeography; in situ and ex situ conservation; nutrigenomics) to generate metadata, will be essential to achieve the sustainable use of understudied and overused plant resources in Sub-Saharan Africa. We believe that this is a key point for further inquiry to respond to some issues raised by the Sustainable Development Goals, such as increasing nutritional wellbeing, food security, and reducing hunger and poverty.

Finally, our study highlights the lack of taxonomic, genetic and ecogeographic knowledge of native *Vigna* and *Phaseolus*, and that greater efforts should be made to improve the in situ and ex situ conservation of these species, especially the more restricted ones. The coordination of national, regional and international conservation strategies is important to ensure the preservation of native edible African flora.

## Methods

### Species diversity, distribution and conservation in Africa

Initial identification of *Vigna* and *Phaseolus* species in Africa was based in Herbarium data from the Instituto Superior de Agronomia of the University of Lisbon, João de Carvalho e Vasconcellos (LISI); Instituto de Investigação Científica Tropical, University of Lisbon (LISC); Museu Nacional de História Natural e da Ciência, University of Lisbon (LISU); University of Coimbra (COI); Royal Botanic Gardens, Kew (K); Natural History Museum (BM); and Meise Botanic Garden (BR).

Online databases were also accessed, namely Plants of the World Online^9^, African Plant Database^74^, Flora of Botswana^75^, Flora of Malawi^76^, Flora of Mozambique^77^, Flora of Zambia^78^, and Flora of Zimbabwe^79^ for information on taxonomic data and native distribution; PROTA—Plant Resources of Tropical Africa^39^, and the International Legume Database and Information Service^80^ were used for information on the cultivation status; the IUCN Red List of Threatened Species^34^ for information on in situ conservation status; and the Genesys Database^35^ to access the ex situ conservation in worldwide gene banks. Finally, scientific publications on *Vigna* and *Phaseolus* and their distribution^14,36,81–83^ were consulted. With the information collected, we constructed a comprehensive dataset that included the scientific name of each taxon, English common names, native status in Africa, native distribution, and conservation category according to the IUCN Red List^34^, and ex situ conservation data.

The geographical distribution of *Vigna* and *Phaseolus* species in Africa was estimated based on occurrence data available on the GBIF database^84^. A total of 47,439 records of *Vigna*^85^ and 10,685 records of *Phaseolus*^86^ were downloaded with geographical coordinates. The location of each record was confirmed at country level, and the records with incorrect location or inaccurate taxonomy were deleted. The final dataset, including 43,257 occurrences of *Vigna* and 8907 occurrences of *Phaseolus* were used in the analysis of species richness (Supplementary Fig. S2). The species richness maps were constructed using a grid of 200 × 200 km and the boundaries of African countries in QGIS v.3.4.4^87^. Finally, the diversity hotspots of *Vigna* genus (i.e. areas with a great number of native species) were identified and compared with the results of previous studies^14^.

### Sampling of *P. vulgaris* and *V. unguiculata*

**Figure 7:**
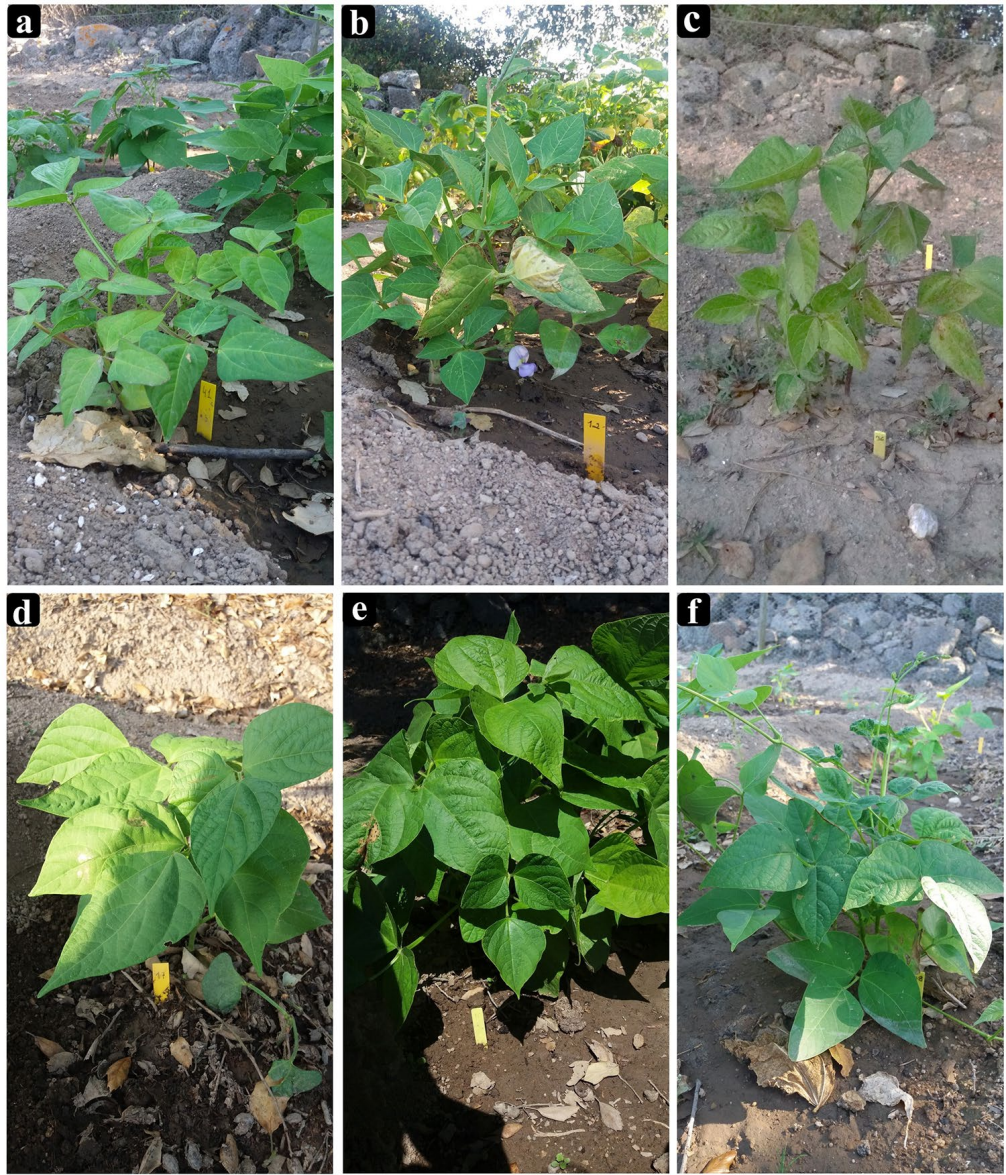
Cultivated samples collected in different African countries: (**a**) *Vigna unguiculata* from Malanje, Angola (MA41Vu); (**b**) *V. unguiculata* from Sofala, Mozambique (SO12Vu); (**c**) *V. unguiculata* from Santiago, Cabo Verde (CV36Vu); (**d**) *Phaseolus vulgaris* from Cunene, Angola (CU17Pv); (**e**) *P. vulgaris* from Sofala, Mozambique (SO10Pv); and (**f**) *P. vulgaris* from Santiago, Cabo Verde (CV38Pv). Plants growth under controlled conditions, between June 2019 and February 2020, at Instituto Superior de Agronomia of the University of Lisbon (ISA/UL).

A total of 38 accessions (Supplementary Table S2) of the most cultivated species, *P. vulgaris* (n = 21) and *V. unguiculata* (n = 17), were collected between 2018 and 2019 in Angola (20), Mozambique (13); and Cabo Verde (5) (Supplementary Fig. S3). Each sample was divided in two parts, one was used for the chemical analysis (n = 38) and the other cultivated to obtain developed and mature leaves for flow cytometric analysis (n = 33). Cultivation took place between June 2019 and February 2020, in a greenhouse under controlled conditions (i.e., using the same kind of well-drained fertile soils in a dimensionally equal pots at an optimal temperature for germination of ± 25 °C) at Instituto Superior de Agronomia of the University of Lisbon (ISA/UL) as shown in the Fig. 7. The germination experiments were performed in triplicate for each accession. Herbarium vouchers were deposited in the Herbarium LISI and the identification of plant specimens was done by some of the authors (SC, MB and MR). Permissions to collect all the seeds used in this study were obtained. This study complies with local and national regulations.

### Physicochemical characterization of *P. vulgaris* and *V. unguiculata*

#### Physical characterization

In order to complement the characterization of the study samples of *P. vulgaris* and *V. unguiculata*, a description of seed phenotypic traits was performed (i.e. quantitative: length, width, and height; and qualitative: shape, color and hilum). For each bean accession, seed length, width, and height were obtained for 10 seeds by using a digital calliper with 0.01 mm of accuracy. Specifically, seed length was measured from the base to the tip portion while the seed width was measured from the hilum to the opposite side. The mean values were recorded in millimeters. The shape and color of the bean, and the description of the hilum was obtained through direct observation of a randomized pool of 10 seeds.

#### Mineral analysis

Mineral contents (Na, K, Ca, Mg, P, S, Fe, Cu, Zn, Mn, B) in *P. vulgaris* and *V. unguiculata* seeds were determined by inductively coupled plasma optical emission spectrometry (ICP-OES). Grain beans were grounded into a powdery form with a stainless-steel grinder (Kunft coffee mill). A portion of 0.3 g from bean powder from each sample was weighed and subjected to the digestion process with a mixture of nitric acid and hydrochloric acid (1:3, v/v) at 105 °C for 90 min and analyzed using the Thermo Scientific iCAP 7000 Series ICP-OES spectrometer (Thermo Scientific, Cambridge, UK). Procedural blanks were obtained following the above-mentioned protocol. Calibration curves of five different concentrations were applied to calculate the concentration of each mineral. Standard solutions of minerals for ICP-OES were from Panreac (Barcelona, Spain) and all the water used was purified using a Milli-Q water system (Millipore, Bedford, MA, USA). All the measurements were carried out in triplicate and the results obtained in mg per kg wet weight.

### Nuclear DNA content estimation

For each cultivated sample (n = 33), young leaves in healthy conditions were randomly collected and immediately analysed in the laboratory. Nuclear DNA content was measured by flow cytometry using *Solanum lycopersicum* ‘Stupické’ [2C = 1.96 pg]^88^ as DNA standard. Each sample was chopped with a razor blade along with the standard in the presence of 1 mL of buffer^89^. The nuclear suspension obtained was filtrated using a 30 μm nylon filter to separate cells from plant debris. After filtration, 50 μg/mL of propidium iodide (PI; Sigma-Aldrich) and 50 μg/mL of RNase (Sigma-Aldrich) were added to stain DNA and prevent staining of double-stranded RNA, respectively. The samples were maintained at room temperature and analyzed using a CyFlow Space flow cytometer (Sysmex, Norderstedt, Germany) as previously described by Guilengue et al*.*^68^. The reproducibility of the results was assessed using five independent replicates for each accession. FloMax software v2.4d (Sysmex) was used to measure nuclear DNA content and three graphics were generated from data measurement: fluorescence pulse integral in linear scale (FL); fluorescence pulse integral in linear scale versus time; and fluorescence pulse integral in linear scale versus side light scatter in logarithmic scale (SSC). The absolute DNA amount of a sample was calculated based on the values of the G1 peak means, as suggested by Doležel and Bartoš^90^:

$$\text{Sample } \; 2 \text{C} \; \text{DNA} \; \text{content}=\frac{\text{Sample} \; \text{G}1 \; \text{peak} \; \text{mean}}{\text{standard} \; \text{G}1 \; \text{ peak} \; \text{mean}}\times \text{Standard } \; 2\text{C} \; \text{DNA} \; \text{Content}$$

The results generated from 2C DNA (in picogram) were transformed to million base pairs using the following conversion: 1 pg = 978 Mbp^91^. Coefficient of variation (CV, %) of G1 peaks in the FL histograms, and estimates of the CV of the genome size of each accession were used to assess the reliability of the results.

### Statistical analysis

All data measurements are presented as mean values. In order to compare morphometric measurements, mineral content, and genome size across the accessions, a Univariate analysis (UA) was performed. Test of means was carried out using Scott Knott test for all variables at 5% significance with the ScottKnott package^92^. To perform a multivariate analysis the mineral content data was standardized (mean = 0, and standard deviation = 1). Cluster analysis was performed based on Euclidean distance and average method for the 38 accessions (heatmap function). Results of cluster analysis were visualized with the ggplot function of ggplot2 package^93^. All analyses were performed in the RStudio program version 1.1.456^94^.

## Supplementary Information

Supplementary Information.

## References

[1] Lewis GP (2005). Legumes of the World.

[2] The Legume Phylogeny Working Group (LPWG) (2017). A new subfamily classification of the Leguminosae based on a taxonomically comprehensive phylogeny. Taxon.

[3] Yahara T (2013). Global legume diversity assessment: Concepts, key indicators, and strategies. Taxon.

[4] Odendo M, Bationo A, Kimani S, Bationo A (2011). Socio-economic contribution of legumes to livelihoods in Sub-Saharan Africa. Fighting Poverty in Sub-Saharan Africa: The Multiple Roles of Legumes in Integrated Soil Fertility Management.

[5] Dakora FD, Keya SO (1997). Contribution of legume nitrogen fixation to sustainable agriculture in Sub-Saharan Africa. Soil Biol. Biochem..

[6] Ajeigde HA, Singh BB, Osenj TO (2005). Cowpea-cereal intercrop productivity in the Sudan savanna zone of Nigeria as affected by planting pattern, crop variety and pest management. Afr. Crop Sci. J..

[7] Rahmanian M, Batello C, Calles T (2018). Pulse Crops for Sustainable Farms in Sub-Saharan Africa.

[8] Rawal V, Navarro DK (2017). The Global Economy of Pulses.

[9] Plants of the World Online. http://powo.science.kew.org (2020).

[10] Broughton WJ (2003). Beans (*Phaseolus* spp.)—Model food legumes. Plant Soil.

[11] Delgado-Salinas A, Bibler R, Lavin M (2006). Phylogeny of the genus *Phaseolus* (Leguminosae): A recent diversification in an ancient landscape. Syst. Bot..

[12] Greenway PJ (1945). Origins of some East African food plants: Part V. East Afr. Agric. J..

[13] Wortmann CS, Allen DJ (1994). African Bean Production Environments: Their Definition, Characteristics and Constraints. Occasional Publication Series 11.

[14] Maxted N (2004). African Vigna: Systematic and Ecogeographic Studies.

[15] Singh BB (2014). Cowpea: The Food Legume of the 21st Century.

[16] Catarino S (2021). Conservation priorities for African *Vigna* species: Unveiling Angola’s diversity hotspots. Glob. Ecol. Conserv..

[17] Vidigal P, Romeiras MM, Monteiro F, Hasanuzzaman M (2019). Crops diversification and the role of orphan legumes to improve the Sub-Saharan Africa farming systems. Sustainable Crop Production.

[18] Maréchal R (1978). Etude taxonomique d'un groupe complexe d'espèces des genres *Phaseolus* et *Vigna* (Papilionaceae) sur la base de données morphologiques et polliniques, traitées par l'analyse informatique. Boissiera.

[19] Peksen E, Peksen A, Gulumser A (2014). Leaf and stomata characteristics and tolerance of cowpea cultivars to drought stress based on drought tolerance indices under rainfed and irrigated conditions. Int. J. Curr. Microbiol. Appl. Sci..

[20] Iqbal A, Khalil IA, Ateeq N, Khan MS (2006). Nutritional quality of important food legumes. Food Chem..

[21] African Orphan Crops Consortium. http://africanorphancrops.org/meet-the-crops/ (2021)

[22] Boukar O, de Ron AM (2015). Cowpea. Grain Legumes.

[23] Animasaun DA, Oyedeji S, Azeez YK, Mustapha OT, Azeez MA (2015). Genetic variability study among ten cultivars of cowpea (*Vigna unguiculata* L. Walp) using morpho-agronomic traits and nutritional composition. J. Agric. Sci..

[24] Timko MP, Singh BB, Moore PH, Ming R (2008). Cowpea, a multifunctional legume. Plant Genetics and Genomics: Crops and Models.

[25] Wortmann SC, Kirkby AR, Eledu AC, Allen JD (2004). Atlas of Common Bean (*Phaseolus vulgaris* L.) Production in Africa.

[26] Guignard MS (2016). Genome size and ploidy influence angiosperm species' biomass under nitrogen and phosphorus limitation. New Phytol..

[27] Sheidai M (2014). Genetic diversity and genome size variability in *Linum austriacum* (Lineaceae) populations. Biochem. Syst. Ecol..

[28] Kron P, Suda J, Husband BC (2007). Applications of flow cytometry to evolutionary and population biology. Annu. Rev. Ecol. Evol. Syst..

[29] Wu YQ (2016). Genetic analyses of Chinese *Cynodon* accessions by flow cytometry and AFLP markers. Crop Sci..

[30] Parida A, Raina SN, Narayan RKJ (1990). Quantitative DNA variation between and within chromosome complements of *Vigna* species (Fabaceae). Genetica.

[31] Nagl W, Treviranus A (1995). A flow cytometric analysis of the nuclear 2C DNA content in 17 *Phaseolus* species (53 genotypes). Bot. Acta.

[32] Barow M, Meister A (2003). Endopolyploidy in seed plants is differently correlated to systematics, organ, life strategy and genome size. Plant Cell Environ..

[33] Lonardi S (2019). The genome of cowpea (*Vigna unguiculata* [L.] Walp.). Plant J..

[34] The IUCN Red List of Threatened Species. Version 2020-2. https://www.iucnredlist.org/ (2020).

[35] Genesys. Plant Genetic Resources Accession. https://www.genesys-pgr.org/ (2021).

[36] Pope GV, Polhill RM (2001). Flora Zambesiaca, part 5.

[37] Tomooka N, Vaughan DA, Moss H, Maxted N (2002). The Asian Vigna: Genus Vigna Subgenus Ceratotropis Genetic Resources.

[38] Debouck DG (1986). Primary diversification of *Phaseolus* in the Americas: Three centers. Plant Genet. Resour. Newsl..

[39] Plant Resources of Tropical Africa. https://www.prota4u.org/database/ (2021).

[40] Linder HP (2014). The evolution of African plant diversity. Front. Ecol. Evol..

[41] Romeiras MM, Figueira R, Duarte MC, Beja P, Darbyshire I (2014). Documenting biogeographical patterns of African timber species using herbarium records: A conservation perspective based on native trees from Angola. PLoS ONE.

[42] Catarino S (2020). Spatial and temporal trends of burnt area in angola: Implications for natural vegetation and protected area management. Diversity.

[43] Catarino S, Duarte MC, Costa E, Carrero PG, Romeiras MM (2019). Conservation and sustainable use of the medicinal Leguminosae plants from Angola. PeerJ.

[44] Romeiras MM (2016). IUCN Red List assessment of the Cape Verde endemic flora: Towards a global strategy for plant conservation in Macaronesia. Bot. J. Linn. Soc..

[45] Gomes AM (2020). Drought response of cowpea (*Vigna unguiculata* (L.) Walp.) landraces at leaf physiological and metabolite profile levels. Environ. Exp. Bot..

[46] The International Institute of Tropical Agriculture (IITA). https://www.iita.org/ (2021)

[47] Fatokun C (2018). Genetic diversity and population structure of a mini-core subset from the world cowpea (*Vigna unguiculata* (L.) Walp.) germplasm collection. Sci. Rep..

[48] Rocha V, Duarte MC, Catarino S, Duarte I, Romeiras MM (2021). Cabo Verde’s Poaceae flora: A reservoir of crop wild relatives diversity for crop improvement. Front. Plant Sci..

[49] Brilhante M (2021). Tackling food insecurity in Cabo Verde Islands: The nutritional, agricultural and environmental values of the legume species. Foods.

[50] Pasquet RS, Pickersgill B, Lock JM (1996). Wild cowpea (*Vigna unguiculata*) evolution. Advances in Legume Systematics 8: Legumes of Economic Importance.

[51] Di Bella G (2016). Mineral composition of some varieties of beans from Mediterranean and Tropical areas. Int. J. Food Sci. Nutr..

[52] Gelin JR, Forster S, Grafton KF, McClean PE, Rojas-Cifuentes GA (2007). Analysis of seed zinc and other minerals in a recombinant inbred population of navy bean (*Phaseolus vulgaris* L.). Crop Sci..

[53] Dakora FD, Belane AK (2019). Evaluation of protein and micronutrient levels in edible cowpea (*Vigna unguiculata* L. Walp) leaves and seeds. Front. Sustain. Food Syst..

[54] Yeken MZ, Akpolat H, Karaköy T, Çiftçi V (2018). Assessment of mineral content variations for biofortification of the bean seed. Int. J. Agric. Sci..

[55] Gondwe TM, Alamu EO, Mdziniso P, Maziya-Dixon B (2019). Cowpea (*Vigna unguiculata* (L.) Walp) for food security: An evaluation of end-user traits of improved varieties in Swaziland. Sci. Rep..

[56] Sperotto RA, Ricachenevsky FK, Williams LE, Vasconcelos MW, Menguer PK (2014). From soil to seed: Micronutrient movement into and within the plant. Front. Plant Sci..

[57] Maziya-Dixon B, Kling JG, Menkir A, Dixon A (2000). Genetic variation in total carotene, iron, and zinc contents of maize and cassava genotypes. Food Nutr. Bull..

[58] Shewfelt RL (1990). Sources of variation in the nutrient content of agricultural commodities from the farm to the consumer. J. Food Qual..

[59] World Health Organization. *The World Health Report 2006: Working Together for Health*. https://www.who.int/whr/2006/whr06_en.pdf?ua=1 (2006).

[60] Gödecke T, Stein AJ, Qaim M (2018). The global burden of chronic and hidden hunger: Trends and determinants. Glob. Food Sec..

[61] Shankar AH, Ryan ET (2020). Mineral deficiencies. Hunter's Tropical Medicine and Emerging Infectious Diseases.

[62] Muthayya S (2013). The global hidden hunger indices and maps: An advocacy tool for action. PLoS ONE.

[63] Joy EJ (2014). Dietary mineral supplies in Africa. Physiol. Plant..

[64] World Health Organization. *World health statistics 2015*. https://apps.who.int/iris/bitstream/handle/10665/170250/9789240694439_eng.pdf;jsessionid=9CFCB446F9217B60415DD216E70F6A49?sequence=1 (2015).

[65] Muriuki JM (2020). Estimating the burden of iron deficiency among African children. BMC Med..

[66] Official Journal of the European Union. *Regulation (Eu) No 1169/2011 of the European Parliament and of the Council of 25 October 2011*. https://eur-lex.europa.eu/legal-content/EN/TXT/PDF/?uri=CELEX:32011R1169&from=EN (2011).

[67] Nowicka A (2016). Nuclear DNA content variation within the genus *Daucus* (Apiaceae) determined by flow cytometry. Sci. Hortic..

[68] Guilengue N, Alves S, Talhinhas P, Neves-Martins J (2020). Genetic and genomic diversity in a tarwi (*Lupinus mutabilis* Sweet) germplasm collection and adaptability to Mediterranean climate conditions. Agronomy.

[69] Chable V (2020). Embedding cultivated diversity in society for agro-ecological transition. Sustainability.

[70] Knight CA, Molinari NA, Petrov DA (2005). The large genome constraint hypothesis: Evolution, ecology and phenotype. Ann. Bot..

[71] Pati K, Zhang F, Batley J (2019). First report of genome size and ploidy of the underutilized leguminous tuber crop Yam Bean (*Pachyrhizus erosus* and *P. tuberosus*) by flow cytometry. Plant Genet. Resour..

[72] Sliwinska E (2018). Flow cytometry—A modern method for exploring genome size and nuclear DNA synthesis in horticultural and medicinal plant species. Folia Hortic..

[73] Veselý P, Bureš P, Šmarda P (2013). Nutrient reserves may allow for genome size increase: Evidence from comparison of geophytes and their sister non-geophytic relatives. Ann. Bot..

[74] African Plant Database. http://www.ville-ge.ch/musinfo/bd/cjb/africa/index. (2021).

[75] Hyde, M. A., Wursten, B. T., Ballings, P. & Coates Palgrave, M. Flora of Botswana. https://www.botswanaflora.com (2021).

[76] Hyde, M. A., Wursten, B. T., Ballings, P. & Coates Palgrave, M. Flora of Malawi. http://www.malawiflora.com (2021).

[77] Hyde, M. A., Wursten, B. T., Ballings, P. & Coates Palgrave, M. Flora of Mozambique. http://www.mozambiqueflora.com (2021)

[78] Bingham, M. G., Willemen, A., Wursten, B. T., Ballings, P. & Hyde, M. A. Flora of Zambia http://www.zambiaflora.com (2021).

[79] Hyde, M. A., Wursten, B. T., Ballings, P. & Coates Palgrave, M. Flora of Zimbabwe. http://www.zimbabweflora.co.zw (2021).

[80] International Legume Database & Information Service. https://ildis.org/LegumeWeb (2020).

[81] Exell, A.W. & Fernandes, A. Conspectus florae angolensis. Vol. 3, No. 2. Leguminosae (Papilionoideae: Hedysareae-Sophoreae) (Junta de Investigações do Ultramar, 1966)

[82] Pasquet RS (2001). Notes on the genus *Vigna* (Leguminosae-Papilionoideae). Kew Bull.

[83] van Zonneveld M (2020). Mapping patterns of abiotic and biotic stress resilience uncovers conservation gaps and breeding potential of *Vigna* wild relatives. Sci. Rep..

[84] Global Biodiversity Information Facility. https://www.gbif.org/ (2021).

[85] GBIF Occurrence Download—*Vigna*. 10.15468/dl.bsjsk5 (2021).

[86] GBIF Occurrence Download—*Phaseolus.*10.15468/dl.kjw72 (2021).

[87] QGIS Development Team. QGIS Geographic Information System. Open Source Geospatial Foundation Project. http://qgis.osgeo.org (2021).

[88] Doležel J, Sgorbati S, Lucretti S (1992). Comparison of three DNA fluorochromes for flow cytometric estimation of nuclear DNA content in plants. Physiol. Plant..

[89] Loureiro J, Rodriguez E, Doležel J, Santos C (2007). Two new nuclear isolation buffers for plant DNA flow cytometry: A test with 37 species. Ann. Bot..

[90] Doležel J, Bartoš J (2005). Plant DNA flow cytometry and estimation of nuclear genome size. Ann. Bot..

[91] Doležel J, Bartoš J, Voglmayr H, Greilhuber J (2003). Nuclear DNA content and genome size of trout and human. Cytometry.

[92] Jelihovschi EG, Faria JC, Allaman IB (2014). ScottKnott: A package for performing the Scott-Knott clustering algorithm in R. TEMA.

[93] Wickham H (2016). ggplot2: Elegant Graphics for Data Analysis.

[94] R Core Team. R: A language and environment for statistical computing https://www.R-project.org/ (R Foundation for Statistical Computing, 2020).

