# Supplementary material for: Exploring physicochemical and cytogenomic diversity of African cowpea and common bean
Source: Sci Rep. 2021 Jun 18;11:12838. doi: 10.1038/s41598-021-91929-2 (PMC8213759; doi:10.1038/s41598-021-91929-2)
Supplement: Supplementary file 1 — Supplementary Information. [file 41598_2021_91929_MOESM1_ESM.docx]

Title: **Exploring physicochemical and cytogenomic diversity of African cowpea and common bean**

Short title: **Diversity patterns of African pulses**

Sílvia Catarino^1,2,*^; Miguel Brilhante^1,3,*^; Anyse Pereira Essoh^1,4,5^; Alberto B. Charrua^1,5,6^; Josefa Rangel^1,7^; Guilherme Roxo^1^; Eromise Varela^1^; Margarida Moldão^1^; Ana Ribeiro-Barros^2^; Salomão Bandeira^8^; Mónica Moura^4^; Pedro Talhinhas^1^; Maria M. Romeiras^1,3**^

^1^Linking Landscape, Environment, Agriculture and Food (LEAF), Instituto Superior de Agronomia (ISA), Universidade de Lisboa, Tapada da Ajuda, 1340-017 Lisboa, Portugal

^2^Forest Research Center (CEF), Instituto Superior de Agronomia (ISA), Universidade de Lisboa, Tapada da Ajuda, 1340-017 Lisboa, Portugal

^3^Centre for Ecology, Evolution and Environmental Changes (cE3c), Faculdade de Ciências, Universidade de Lisboa, Campo Grande, 1749-016 Lisboa, Portugal

^4^Research Centre in Biodiversity and Genetic Resources (CIBIO), InBIO Associate Laboratory, Faculdade de Ciências e Tecnologia, Universidade dos Açores, Ponta Delgada, Portugal

^5^Nova School of Business and Economics, Universidade Nova de Lisboa, Campus de Carcavelos, Rua da Holanda, n.1, Carcavelos, 2775-405 Cascais, Portugal

^6^Department of Earth Sciences and Environment, Faculty of Science and Technology, Licungo University, P.O. Box 2025, Beira 2100, Mozambique

^7^Centro de Botânica, Universidade Agostinho Neto, Luanda, Angola

^8^Department of Biological Sciences, Eduardo Mondlane University, PO Box 257, Maputo 1100, Mozambique

* Equal contribution as first authors

**Corresponding author: mmromeiras@isa.ulisboa.pt

**Figure S1.** Photographs of the 38 *Phaseolus vulgaris* (Pv) and *Vigna unguiculata* (Vu) accessions used in this study, collected, between 2018 and 2019, in Angola, Mozambique and Cabo Verde. For more details on samples origin (country/region); shape, colour and hilum see Table S2.

**
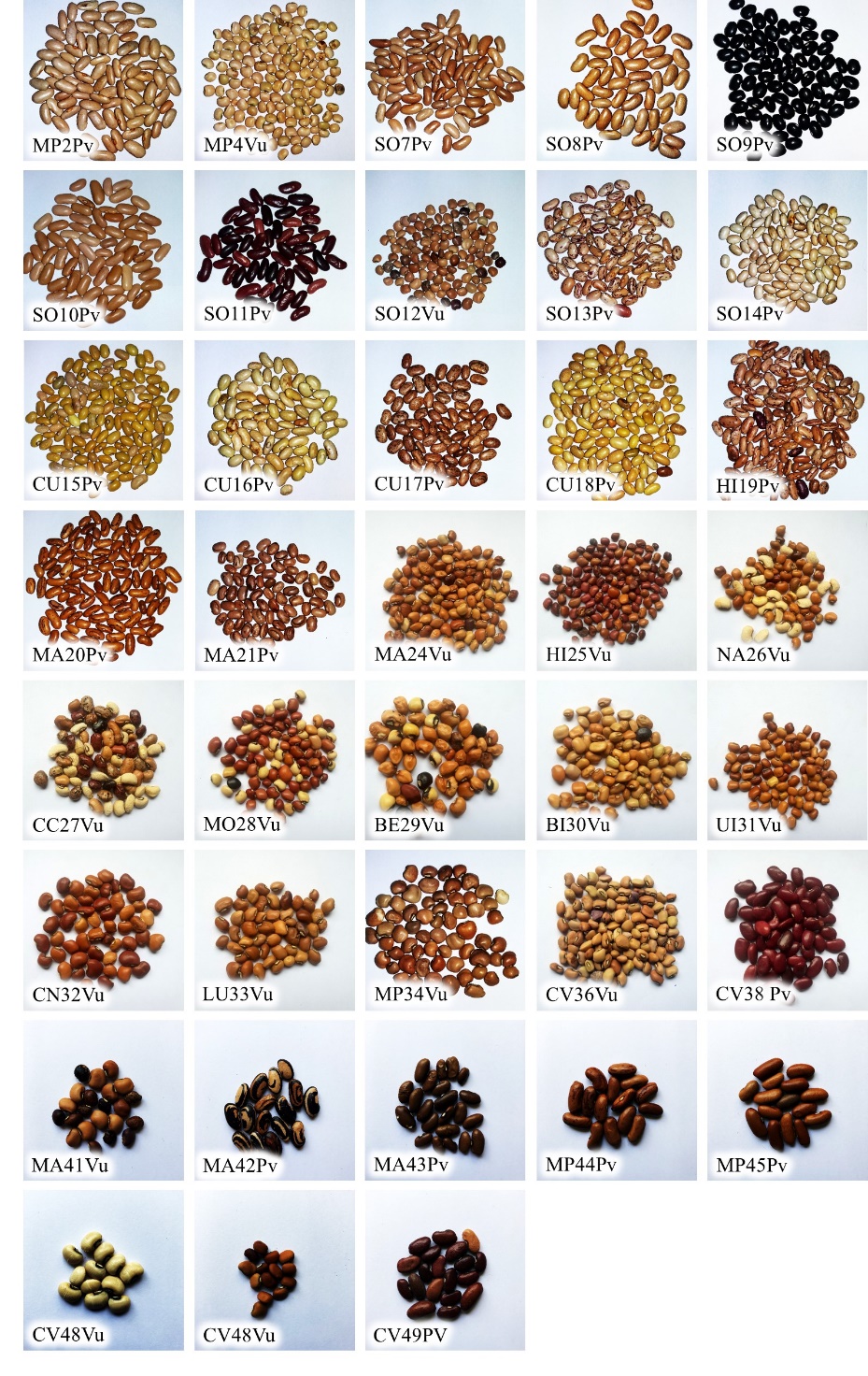
**

**Figure S2.** Occurrence records of **(a)** *Vigna* and **(b)** *Phaseolus* in Africa, available on GBIF [83,84]. This figure was produced with the free available on-line software QGIS v.3.4.4 (http://qgis.osgeo.org).

**
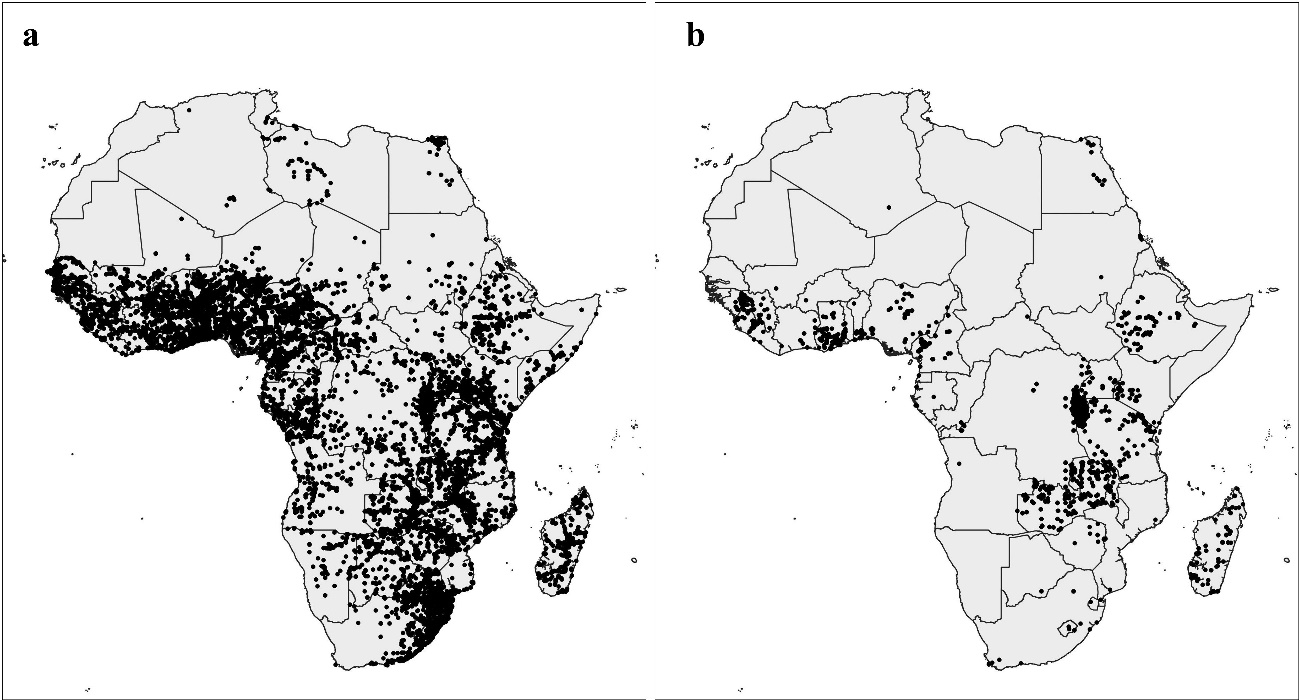
**

**Figure S3.** Countries and provinces where samples were collected (in red colour): Cabo Verde (Santiago), Angola (Uíge, Cuanza Norte, Malanje, Benguela, Namibe, Bié, Moxico, Huíla, Cunene, and Cuando Cubango), and Mozambique (Sofala and Maputo). This figure was produced with the free available on-line software QGIS v.3.4.4 (http://qgis.osgeo.org).


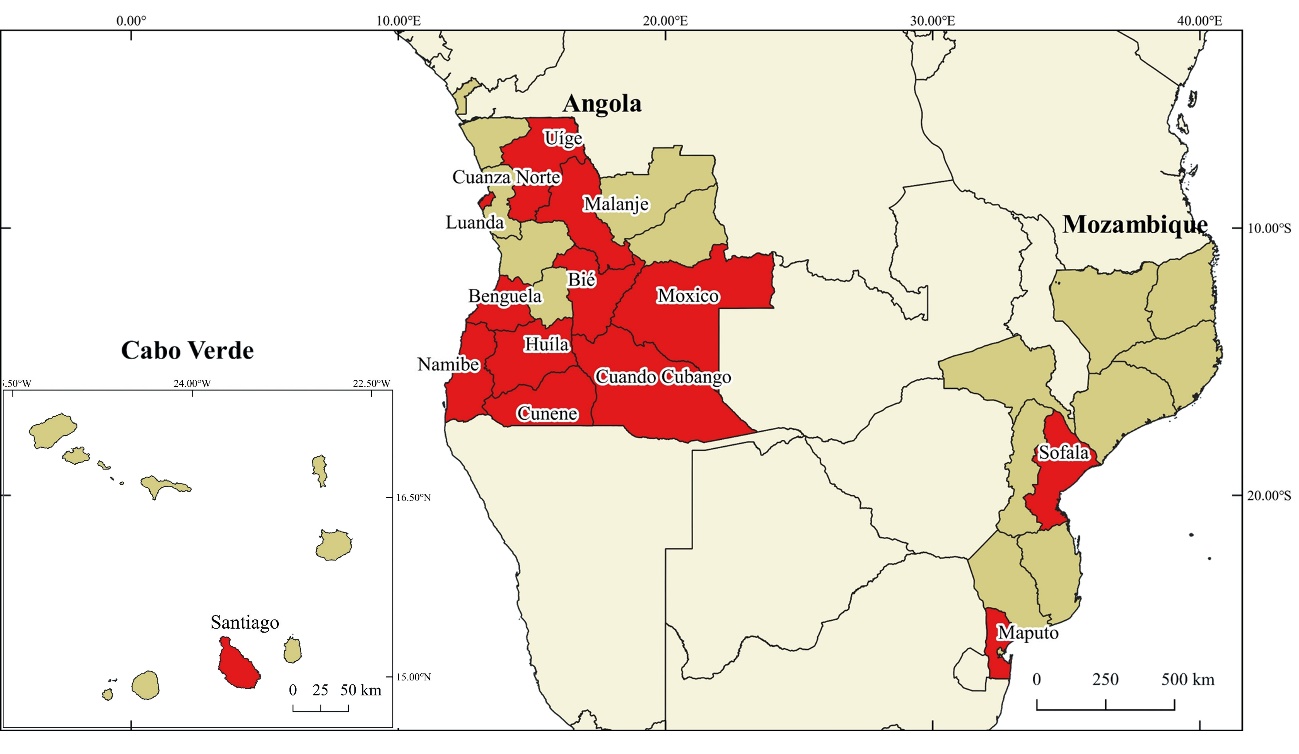


**Table S1.** *Phaseolus* and *Vigna* species occurring in Africa: Scientific name, English common name, status in Africa, known cultivation in Africa, native distribution, and *in situ* and *ex situ* conservation status.

| **Taxon** | **Common names** | **Status in Africa** | **Cultivated in Africa** | **Native distribution** | ***In situ* conservation ^a^** | ***Ex situ* conservation ^b^** | | | |
| --- | --- | --- | --- | --- | --- | --- | --- | --- | --- |
|  |  |  |  |  |  | Number of accessions in Angola | Number of cessions in Mozambique | Number of accessions in Cabo Verde | Available worldwide accessions |
| ***Phaseolus*** |  |  |  |  |  |  |  |  |  |
| *Phaseolus acutifolius* A.Gray | Tepary Bean | Introduced | Yes | Arizona to Texas and Mexico | LC | 0 | 0 | 0 | 1439 |
| *Phaseolus coccineus* L. | Runner Bean | Introduced | Yes | Mexico to Central America | LC | 0 | 0 | 0 | 5015 |
| *Phaseolus lunatus* L. | Lima Bean | Introduced | Yes | Central America | LC | 2 | 0 | 1 | 8076 |
| *Phaseolus massaiensis*Taub. |  | Native |  | Tanzania | NE | 0 | 0 | 0 | 0 |
| *Phaseolus vulgaris* L. | Common Bean | Introduced | Yes | Central America | LC | 290 | 0 | 1 | 136 167 |
| ***Vigna*** |  |  |  |  |  |  |  |  |  |
| *Vigna aconitifolia* (Jacq.) Maréchal | Dew-Bean, Moth Bean | Introduced | Yes | Indian Subcontinent to China | DD | 0 | 0 | 0 | 287 |
| *Vigna ambacensis* Welw. ex Baker | Wild vigna | Native |  | Tropical Africa | LC | 0 | 0 | 0 | 242 |
| *Vigna angivensis* Baker | Wild vigna | Native |  | Madagascar | LC | 0 | 0 | 0 | 6 |
| *Vigna angularis* (Willd.) Ohwi & H.Ohashi | Adzuki Bean | Introduced | Yes | Nepal to China, Taiwan and Japan | LC | 0 | 0 | 0 | 3316 |
| *Vigna antunesii* Harms |  | Native |  | Tanzania to Tropical Africa | LC | 0 | 0 | 0 | 0 |
| *Vigna bequaertii* R.Wilczek |  | Native |  | Central Tropical Africa | NE | 0 | 0 | 0 | 0 |
| *Vigna bosseri* Du Puy & Labat |  | Native |  | Madagascar | EN | 0 | 0 | 0 | 0 |
| *Vigna comosa* Baker |  | Native |  | Tropical Africa | LC | 0 | 0 | 0 | 13 |
| *Vigna debanensis* Martelli |  | Native |  | Eritrea to N. Ethiopia | NE | 0 | 0 | 0 | 0 |
| *Vigna dolomitica* R.Wilczek |  | Native |  | Democratic Republic of the Congo | CR | 0 | 0 | 0 | 1 |
| *Vigna filicaulis* Hepper |  | Native |  | Tropical Africa to Chad | LC | 0 | 0 | 0 | 14 |
| *Vigna fischeri* Harms |  | Native |  | Ethiopia to Zambia | LC | 0 | 0 | 0 | 1 |
| *Vigna friesiorum* Harms |  | Native |  | Ethiopia to Central Tropical Africa | LC | 0 | 0 | 0 | 11 |
| *Vigna frutescens* A.Rich. | Wild sweetpea | Native |  | Tropical and southern Africa | LC | 0 | 1 | 0 | 44 |
| *Vigna gazensis* Baker f. |  | Native |  | Tropical Africa and Madagascar | LC | 0 | 0 | 0 | 0 |
| *Vigna gracilis* (Guill. & Perr.) Hook.f. | Wild vigna | Native |  | West Tropical Africa | LC | 0 | 0 | 0 | 49 |
| *Vigna haumaniana* R.Wilczek |  | Native |  | Tanzania to Zambia | NE | 0 | 0 | 0 | 0 |
| *Vigna heterophylla* A.Rich. | Wild vigna | Native |  | Tropical Africa | NE | 0 | 0 | 0 | 31 |
| *Vigna hosei* (Craib) Backer | Sarawak Bean | Introduced | Yes | Borneo, Jawa, Malaya and Taiwan | DD | 0 | 0 | 0 | 65 |
| *Vigna jaegeri*Harms |  | Native |  | Tanzania | NE | 0 | 0 | 0 | 0 |
| *Vigna juncea* Milne-Redh. |  | Native |  | Tropical Africa | LC | 0 | 0 | 0 | 0 |
| *Vigna juruana* (Harms) Verdc. |  | Introduced |  | Central and Tropical America | DD | 0 | 0 | 0 | 3 |
| *Vigna keraudrenii* Du Puy & Labat |  | Native |  | Madagascar | EN | 0 | 0 | 0 | 0 |
| *Vigna kirkii*(Baker) J.B.Gillett |  | Native |  | Tropical Africa | LC | 0 | 0 | 0 | 9 |
| *Vigna kokii* B.J.Pienaar |  | Native |  | Namibia and northern provinces of South Africa | LC | 0 | 0 | 0 | 1 |
| *Vigna laurentii* De Wild. |  | Native |  | Tropical Africa | EN | 0 | 0 | 0 | 6 |
| *Vigna lobatifolia* Baker | Wild vigna | Native |  | Tropical Africa to Namibia | NE | 0 | 0 | 0 | 4 |
| *Vigna longifolia* (Benth.) Verdc. |  | Introduced |  | Mexico to South Tropical America | LC | 0 | 0 | 0 | 17 |
| *Vigna longissima* Hutch. |  | Native |  | Nigeria to Tanzania and Zambia | NE | 0 | 0 | 0 | 0 |
| *Vigna luteola* (Jacq.) Benth. | Hairypod cowpea | Native | Yes | Tropics and subtropics (worldwide) | LC | 0 | 0 | 0 | 277 |
| *Vigna marina* (Burm.) Merr. | Beach Bean | Native | Yes | Tropical and Subtropical Africa and Asia, Australia | LC | 0 | 2 | 0 | 131 |
| *Vigna membranacea* A.Rich. | Wild vigna | Native |  | Eritrea to Tanzania and Southwest Arabian Peninsula | LC | 0 | 0 | 0 | 89 |
| *Vigna mendesii* Torre |  | Native |  | Angola | NE | 0 | 0 | 0 | 0 |
| *Vigna microsperma* R.Vig. |  | Native |  | Madagascar | LC | 0 | 0 | 0 | 0 |
| *Vigna mildbraedii*Harms |  | Native |  | Central Rwanda | NE | 0 | 0 | 0 | 0 |
| *Vigna monantha*Thulin |  | Native |  | Central Somalia | EN | 0 | 0 | 0 | 0 |
| *Vigna monophylla*Taub. |  | Native |  | Ethiopia to Botswana | LC | 0 | 0 | 0 | 8 |
| *Vigna mudenia* B.J.Pienaar |  | Native |  | South Africa | NE | 0 | 0 | 0 | 1 |
| *Vigna multinervis* Hutch. & Dalziel | Wild vigna | Native |  | Tropical Africa | LC | 0 | 0 | 0 | 23 |
| *Vigna mungo* (L.) Hepper | Black Gram | Introduced | Yes | Indian Subcontinent | NE | 0 | 0 | 0 | 1647 |
| *Vigna nervosa*Markötter |  | Native |  | Zimbabwe to South Africa | NE | 0 | 0 | 0 | 52 |
| *Vigna nigritia*Hook.f. |  | Native |  | West and central Africa | LC | 0 | 0 | 0 | 34 |
| *Vigna nuda* N.E.Br. |  | Native |  | Tanzania to Zimbabwe | NE | 0 | 0 | 0 | 0 |
| *Vigna nyangensis*Mithen |  | Native |  | Zimbabwe and Angola | LC | 0 | 0 | 0 | 1 |
| *Vigna oblongifolia* A.Rich. |  | Native |  | Tropical and southern Africa, and Madagascar | LC | 0 | 0 | 0 | 276 |
| *Vigna parkeri* Baker | Creeping vigna | Native | Yes | Tropical Africa and Madagascar | LC | 0 | 0 | 0 | 77 |
| *Vigna phoenix* Brummitt |  | Native |  | Tanzania to Zambia | NE | 0 | 0 | 0 | 1 |
| *Vigna platyloba*Welw. ex Hiern |  | Native |  | Tanzania to Tropical Africa | LC | 0 | 0 | 0 | 5 |
| *Vigna procera* Welw. ex Hiern |  | Native |  | Angola and Zambia | NT | 0 | 0 | 0 | 0 |
| *Vigna pseudovenulosa* (Maréchal, Mascherpa & Stainier) Pasquet & Maesen |  | Native |  | Senegal to Central African Republic | NE | 0 | 0 | 0 | 0 |
| *Vigna pubigera* Baker |  | Native |  | Tropical Africa | NE | 0 | 0 | 0 | 0 |
| *Vigna pygmaea* R.E.Fr. |  | Native |  | Cameroon to Tanzania and Botswana | LC | 0 | 0 | 0 | 0 |
| *Vigna racemosa* (G.Don) Hutch. & Dalziel ex Baker f. | Wild vigna | Native |  | Tropical Africa to Namibia | LC | 0 | 0 | 0 | 201 |
| *Vigna radiata* (L.) R.Wilczek | Mung Bean | Introduced | Yes | Arabian Peninsula, Taiwan to Tropical Asia and Australia | LC | 2 | 0 | 0 | 15 944 |
| *Vigna radicans* Welw. ex Baker |  | Native |  | Nigeria to Kenya and Tropical Africa | NE | 0 | 0 | 0 | 6 |
| *Vigna ramanniana*Rossbach |  | Native |  | Angola | NE | 0 | 0 | 0 | 0 |
| *Vigna reticulata* Hook.f. | Wild vigna | Native |  | Tropical Africa and Madagascar | LC | 0 | 0 | 0 | 186 |
| *Vigna richardsiae* Verdc. |  | Native |  | Tanzania to Zambia | DD | 0 | 0 | 0 | 0 |
| *Vigna schimperi* Baker | Wild vigna | Native |  | Ethiopia to Malawi | LC | 0 | 0 | 0 | 33 |
| *Vigna schlechteri*Harms |  | Native |  | Mozambique, Zimbabwe and Northern South Africa | LC | 0 | 0 | 0 | 0 |
| *Vigna somaliensis* Baker f. |  | Native |  | Somalia | DD | 0 | 0 | 0 | 0 |
| *Vigna stenophylla* Harms |  | Native |  | West and central Tropical Africa | NE | 0 | 0 | 0 | 0 |
| *Vigna subterranea* (L.) Verdc. | Bambara Bean, Bambara Groundnut | Native | Yes | Cameroon, Central African Republic, Chad, Nigeria, and Sudan | LC | 16 | 1 | 0 | 2541 |
| *Vigna tisserantiana* Pellegr. |  | Native |  | Central African Republic | DD | 0 | 0 | 0 | 0 |
| *Vigna trichocarpa* (C.Wright) A.Delgado |  | Introduced |  | Mexico to Tropical America | NE | 0 | 0 | 0 | 0 |
| *Vigna trilobata* (L.) Verdc. | African Gram | Introduced |  | Indian Subcontinent to China and Indo-China | NE | 0 | 0 | 0 | 191 |
| *Vigna triphylla* (R.Wilczek) Verdc. |  | Native |  | Tropical Africa | NE | 0 | 0 | 0 | 3 |
| *Vigna umbellata* (Thunb.) Ohwi & H.Ohashi | Rice Bean | Introduced | Yes | Tropical and Subtropical Asia | NE | 0 | 0 | 0 | 928 |
| *Vigna unguiculata* (L.) Walp. | Cowpea, Black Eye Pea | Native | Yes | Tropical and southern Africa | NE | 217 | 46 | 0 | 40 368 |
| *Vigna venulosa* Baker |  | Native |  | Tropical Africa to Chad | NE | 0 | 0 | 0 | 5 |
| *Vigna verdcourtii* Pasquet |  | Native |  | Ethiopia, Malawi, Tanzania and Zambia | NE | 0 | 0 | 0 | 0 |
| *Vigna vexillata* (L.) A.Rich. | Zombi-Pea | Native | Yes | Tropics and subtropics (worldwide) | NE | 0 | 8 | 0 | 952 |
| *Vigna wittei* Baker f. |  | Native |  | Nigeria to Tanzania and Namibia | NE | 0 | 0 | 0 | 30 |

**^a^ *In situ* conservation:** conservation status assessed through IUCN Red List (**CR**, Critically Endangered; **EN**, Endangered; **VU**, Vulnerable; **NT**, Near threatened; **LC**, Least concern; **DD**, Data deficient; **NE**, Not evaluated).

^b^ ***Ex situ* conservation**: Accessions in worldwide genebanks assessed through the Genesys Database (Genesys, 2021).

**Table S2.** List of the 38 *Phaseolus vulgaris* and *Vigna unguiculata* accessions used for this study: origin (country/region); shape, color and hilum.

| **Accession** | **Species** | **Origin** | **Shape** | **Color** | **Hilum** |
| --- | --- | --- | --- | --- | --- |
| MP02Pv | *Phaseolus vulgaris* L. | Mozambique, Maputo | Oblong | Pale Brown | White with a brown rim |
| MP04Vu | *Vigna unguiculata* (L.) Walp. | Mozambique, Maputo | Globose | Pearl | White with a yellow rim |
| SO07Pv | *Phaseolus vulgaris* L. | Mozambique, Sofala | Oblong | Brown | White with a brown rim |
| SO08Pv | *Phaseolus vulgaris* L. | Mozambique, Sofala | Oblong | Brown | White with a brown rim |
| SO09Pv | *Phaseolus vulgaris* L. | Mozambique, Sofala | Oblong or Globose | Black | White with a black rim |
| SO10Pv | *Phaseolus vulgaris* L. | Mozambique, Sofala | Oblong | Pale Brown | White with a brown rim |
| SO11Pv | *Phaseolus vulgaris* L. | Mozambique, Sofala | Oblong | Dark Brown | White with a dark brown rim |
| SO12Vu | *Vigna unguiculata* (L.) Walp. | Mozambique, Sofala | Globose | From Pale to Dark Brown and some with black spots | White with a black rim |
| SO13Pv | *Phaseolus vulgaris* L. | Mozambique, Sofala | Oblong | Pale brown with dark brown spots | White with a brown rim |
| SO14Pv | *Phaseolus vulgaris* L. | Mozambique, Sofala | Oblong | White | White with a white rim |
| CU15Pv | *Phaseolus vulgaris* L. | Angola, Cunene | Oblong | Yellow | White with a brown rim |
| CU16Pv | *Phaseolus vulgaris* L. | Angola, Cunene | Oblong | Pale yellow | White with a black rim |
| CU17Pv | *Phaseolus vulgaris* L. | Angola, Cunene | Oblong | Pale brown with dark brown spots | White with a brown rim |
| CU18Pv | *Phaseolus vulgaris* L. | Angola, Cunene | Oblong | Yellow to pale yellow | White with a black rim |
| HI19Pv | *Phaseolus vulgaris* L. | Angola, Huíla | Oblong | Pale brown with dark brown spots | White with a brown rim |
| MA20Pv | *Phaseolus vulgaris* L. | Angola, Malanje | Oblong | Pale brown with dark brown spots | White with a brown rim |
| MA21Pv | *Phaseolus vulgaris* L. | Angola, Malanje | Oblong | Pale brown with dark brown spots | White with a brown rim |
| MA24Vu | *Vigna unguiculata* (L.) Walp. | Angola, Malanje | Globose | Pale brown | White with a black rim |
| HI25Vu | *Vigna unguiculata* (L.) Walp. | Angola, Huíla | Globose | Pale brown to reddish brown | White with a brown rim |
| NA26Vu | *Vigna unguiculata* (L.) Walp. | Angola, Namibe | Globose | Pale brown to dark brown | White with a black rim |
| CC27Vu | *Vigna unguiculata* (L.) Walp. | Angola, Cuando Cubango | Globose | Pearl | White with a black rim |
| MO28Vu | *Vigna unguiculata* (L.) Walp. | Angola, Moxico | Globose | White or brown | White with a black rim |
| BE29Vu | *Vigna unguiculata* (L.) Walp. | Angola, Benguela | Globose | White, pale brown or black some with dark spots | White with a black rim |
| BI30Vu | *Vigna unguiculata* (L.) Walp. | Angola, Bié | Globose | Brown to pale brown | White with a black rim |
| UI31Vu | *Vigna unguiculata* (L.) Walp. | Angola, Uíge | Globose | Pale brown | White with a brown rim |
| CN32Vu | *Vigna unguiculata* (L.) Walp. | Angola, Cuanza Norte | Globose | Pale brown or reddish brown | White with a black rim |
| LU33Vu | *Vigna unguiculata* (L.) Walp. | Angola, Luanda | Globose | Brown to dark brown | White with a black rim |
| MP34Vu | *Vigna unguiculata* (L.) Walp. | Mozambique, Maputo | Globose | Brown to dark brown | White with a black rim |
| CV36Vu | *Vigna unguiculata* (L.) Walp. | Cabo Verde, Santiago | Globose | Brown to pale brown | White with a brown rim |
| CV38Pv | *Phaseolus vulgaris* L. | Cabo Verde, Santiago | Oblong | Reddish brown | White with a black rim |
| MA41Vu | *Vigna unguiculata* (L.) Walp. | Angola, Malanje | Globose | From Pale to Dark Brown and some with black spots | White with a black or brown rim |
| MA42Pv | *Phaseolus vulgaris* L. | Angola, Malanje | Oblong | Pale brown with black spots | White with a black rim |
| MA43Pv | *Phaseolus vulgaris* L. | Angola, Malanje | Oblong | Brown | White with a black rim |
| MP44Pv | *Phaseolus vulgaris* L. | Mozambique, Maputo | Oblong | Pale brown with brown to reddish brown spots | White with a brown rim |
| MP45Pv | *Phaseolus vulgaris* L. | Mozambique, Maputo | Oblong | Pale brown | White with a brown rim |
| CV47Vu | *Vigna unguiculata* (L.) Walp. | Cabo Verde, Santiago | Oblong | Pearl | White with a brown rim |
| CV48Vu | *Vigna unguiculata* (L.) Walp. | Cabo Verde, Santiago | Globose | Pale brown | White with a brown rim |
| CV49Pv | *Phaseolus vulgaris* L. | Cabo Verde, Santiago | Oblong | Reddish brown | White with a black rim |

**Table S3**. Average values^a^ (mm) and homogeneous groups^b^ for morphometric measurements of 21 *Phaseolus vulgaris* and 17 *Vigna unguiculata* accessions from African countries.

| **Accession** | **Length** | **Width** | **Height** |
| --- | --- | --- | --- |
| SO11Pv | 17.0 a | 7.4 b | 5.9 a |
| MP44Pv | 16.8 a | 7.5 b | 6.7 a |
| SO10Pv | 16.3 a | 7.4 b | 5.8 a |
| SO07Pv | 15.9 b | 7.4 b | 5.9 a |
| MP45Pv | 15.5 b | 6.9 c | 5.4 a |
| MA20Pv | 15.4 b | 6.8 c | 5.9 a |
| CV49Pv | 15.4 b | 6.8 c | 5.4 a |
| MP02Pv | 14.3 c | 7.3 b | 10.1 a |
| HI19Pv | 14.0 c | 7.5 b | 5.6 a |
| SO08Pv | 13.8 c | 6.6 d | 5.3 a |
| SO13Pv | 13.6 c | 7.8 b | 6.1 a |
| MA42Pv | 13.2 d | 6.6 d | 4.5 a |
| CU17Pv | 12.7 d | 7.4 b | 5.3 a |
| CU16Pv | 12.1 e | 6.8 c | 5.9 a |
| SO09Pv | 11.9 e | 8.5 a | 6.8 a |
| SO14Pv | 11.6 f | 7.2 b | 6.0 a |
| CU18Pv | 11.5 f | 7.5 b | 6.6 a |
| MA43Pv | 11.4 f | 6.3 d | 4.9 a |
| CU15Pv | 11.0 f | 7.0 c | 5.9 a |
| CV38Pv | 10.6 g | 6.5 d | 4.3 a |
| MA21Pv | 10.1 g | 6.4 d | 5.0 a |
| Mean ± stDev^c^ | 13.5±2.3 | 7.1±0.7 | 5.9±3.4 |
| CC27Vu | 10.3 a | 7.6 b | 5.5 a |
| CN32Vu | 9.4 b | 7.6 b | 5.2 b |
| NA26Vu | 8.6 c | 6.4 d | 5.1 b |
| MP34Vu | 8.5 c | 8.2 a | 5.9 a |
| BE29Vu | 8.5 c | 6.8 d | 5.7 a |
| SO12Vu | 8.5 c | 7.8 b | 5.8 a |
| MA41Vu | 8.3 c | 7.0 c | 4.8 c |
| CV36Vu | 8.0 d | 6.1 e | 4.5 c |
| BI30Vu | 7.8 d | 5.9 e | 4.4 c |
| HI25Vu | 7.6 d | 5.9 e | 4.3 c |
| MP04Vu | 7.5 d | 6.1 e | 5.0 b |
| LU33Vu | 7.4 d | 5.9 e | 4.5 c |
| CV47Vu | 7.4 d | 6.1 e | 5.1 b |
| MA24Vu | 7.4 d | 6.1 e | 4.4 c |
| MO28Vu | 7.4 d | 6.0 e | 4.6 c |
| CV48Vu | 7.3 d | 6.3 d | 4.7 c |
| UI31Vu | 5.5 e | 4.7 f | 3.2 d |
| Mean ± stDev^c^ | 8.0±1.2 | 6.5±1.0 | 4.9±0.8 |

^a^ Average values of three separate determinations (n=10); ^b^ Homogeneous groups: accessions sharing the same letter for each mineral are not statistically diﬀerent according to the Scott-Knott test at 5% of confidence; ^c^ Standard deviation;

**Table S4.** Average values^a^ (mg/kg ww) and homogeneous groups^b^ for mineral content of 21 *Phaseolus vulgaris* and 17 *Vigna unguiculata* accessions from African countries.

| **Accession** | **Na** | **K** | **Ca** | **Mg** | **P** | **S** | **Fe** | **Cu** | **Zn** | **Mn** | **B** |
| --- | --- | --- | --- | --- | --- | --- | --- | --- | --- | --- | --- |
| MP02Pv | 260.4 a | 10506.7 e | 1063.0 g | 1710.0 e | 4863.9 b | 1783.1 c | 64.7 f | 8.1 b | 31.9 a | 15.3 o | 5.9 c |
| SO09Pv | 235.0 b | 9157.1 g | 1391.2 d | 1732.6 e | 4302.1 f | 1304.9 i | 53.5 j | 7.1 c | 17.5 m | 25.9 b | 0.7 h |
| MA42Pv | 197.1 c | 10493.3 e | 1319.5 d | 1811.8 c | 3908.6 i | 1874.9 b | 58.7 h | 9.9 a | 26.0 d | 24.7 e | 2.8 f |
| SO14Pv | 195.3 c | 8490.8 h | 1701.8 a | 1672.3 f | 3748.5 j | 1786.3 c | 68.7 d | 8.3 b | 21.1 j | 26.1 b | 1.7 g |
| CU15Pv | 190.7 c | 8626.0 h | 1249.2 e | 1640.6 f | 3119.9 l | 1798.0 c | 68.9 d | 8.2 b | 24.3 e | 28.3 a | 2.4 g |
| SO11Pv | 185.0 c | 9121.7 g | 964.3 h | 1492.5 h | 4260.3 f | 1564.1 f | 66.1 e | 7.0 c | 22.9 h | 21.1 i | 3.8 e |
| MP44Pv | 174.8 c | 9617.2 f | 1441.3 c | 1696.3 e | 4046.1 h | 1699.4 d | 79.2 a | 6.0 c | 23.6 g | 25.2 d | 1.3 h |
| CU17Pv | 169.6 c | 9241.0 g | 933.5 h | 1703.3 e | 3878.7 i | 2021.2 a | 56.1 i | 8.8 b | 24.6 e | 18.5 k | 3.9 e |
| MA20Pv | 168.9 c | 9379.5 g | 1379.6 d | 1536.8 h | 3278.7 k | 1544.6 g | 67.4 d | 6.9 c | 22.5 h | 21.8 h | 3.4 e |
| SO10Pv | 161.0 d | 8749.1 h | 1384.7 d | 1602.1 g | 4389.0 e | 1530.6 g | 73.6 b | 7.5 c | 27.1 c | 20.5 j | 1.7 g |
| SO08Pv | 152.5 d | 8646.5 h | 1158.2 f | 1670.5 f | 4712.5 c | 1377.7 h | 70.5 c | 8.0 b | 24.0 f | 20.5 j | 4.8 d |
| MA43Pv | 147.8 d | 10421.6 e | 1040.9 g | 1768.8 d | 4196.0 g | 1732.8 d | 53.0 j | 7.7 b | 23.4 g | 25.6 c | 3.5 e |
| MP45Pv | 137.3 d | 9638.5 f | 1453.6 c | 1636.3 g | 4210.7 g | 1294.7 i | 68.7 d | 7.3 c | 25.9 d | 23.5 f | 2.0 g |
| CV49Pv | 136.3 d | 11845.5 a | 1452.4 c | 1818.9 c | 4196.5 g | 1901.6 b | 79.5 a | 6.8 c | 19.9 l | 17.9 l | 5.9 c |
| HI19Pv | 123.2 e | 10969.4 c | 1446.5 c | 1766.8 d | 3853.6 i | 1974.7 a | 72.6 b | 7.3 c | 28.3 b | 18.5 k | 7.7 b |
| SO07Pv | 121.2 e | 9338.8 g | 1006.3 g | 1515.3 h | 3943.4 i | 1501.4 g | 50.6 k | 6.8 c | 21.8 i | 13.8 q | 5.8 c |
| CU16Pv | 120.9 e | 10691.7 d | 1244.8 e | 1804.6 c | 4059.3 h | 1573.0 f | 62.2 g | 7.2 c | 24.7 e | 18.5 k | 7.5 b |
| CV38Pv | 118.2 e | 11563.9 b | 1384.7 d | 1777.8 d | 4544.1 d | 1817.9 c | 72.4 b | 6.5 c | 23.5 g | 14.5 p | 8.1 b |
| SO13Pv | 109.2 e | 10453.0 e | 1067.1 g | 1726.0 e | 5210.8 a | 1571.4 f | 68.0 d | 8.0 b | 25.6 d | 15.8 n | 7.5 b |
| MA21Pv | 106.8 e | 10449.8 e | 1536.9 b | 1906.4 a | 3814.9 j | 1875.4 b | 49.0 l | 8.0 b | 21.9 i | 22.6 g | 9.4 a |
| CU18Pv | 103.0 e | 10537.9 e | 1214.9 e | 1858.6 b | 4077.3 h | 1626.7 e | 57.6 h | 6.4 c | 20.4 k | 16.7 m | 8.1 b |
| Mean ± stDev ^c^ | 157.8±43.4 | 9901.9±962.4 | 1277.8±205.6 | 1707.0±109.9 | 4124.5±468.4 | 1674.0±206.3 | 64.8±8.8 | 7.5±1.0 | 23.9±3.0 | 20.7±4.2 | 4.7±2.6 |
| LU33Vu | 252.2 a | 10458.8 b | 924.2 c | 2037.6 d | 4543.6 d | 1849.9 e | 58.0 b | 8.2 a | 36.1 c | 23.0 g | 7.5 f |
| MP04Vu | 239.9 a | 9187.5 g | 846.5 d | 1950.7 f | 3622.3 g | 2015.0 c | 39.1 i | 4.7 d | 33.4 e | 19.2 l | 10.5 d |
| CN32Vu | 236.5 a | 10022.0 d | 761.0 e | 2074.8 d | 4082.9 e | 2348.0 a | 58.7 b | 5.4 c | 34.3 d | 21.0 j | 9.7 d |
| NA26Vu | 234.4 a | 10009.7 d | 1006.0 b | 2086.7 d | 5117.9 a | 1896.3 d | 55.4 d | 6.8 b | 35.8 c | 22.4 h | 11.1 c |
| HI25Vu | 194.4 b | 10222.5 c | 717.1 f | 2169.4 c | 4706.5 c | 1918.9 d | 78.0 a | 7.3 b | 28.2 i | 38.0 a | 9.0 e |
| MA24Vu | 190.4 b | 9969.7 d | 852.6 d | 2210.0 b | 4493.8 d | 1720.6 g | 54.0 d | 6.1 c | 26.0 j | 28.8 c | 6.8 f |
| MO28Vu | 177.6 b | 9090.8 g | 829.9 d | 2148.3 c | 3493.9 h | 1873.6 e | 49.6 f | 6.4 b | 28.7 i | 22.1 i | 10.2 d |
| MP34Vu | 171.9 b | 9730.3 e | 851.4 d | 1925.4 f | 3936.5 f | 1867.7 e | 49.4 f | 5.8 c | 29.1 h | 21.0 j | 10.4 d |
| CV36Vu | 171.2 b | 9768.3 e | 796.3 e | 1935.2 f | 5116.1 a | 1950.8 d | 52.9 e | 4.7 d | 29.8 g | 14.8 n | 11.6 c |
| CV48Vu | 169.4 b | 10280.4 c | 908.2 c | 2016.8 e | 4019.3 e | 1958.3 d | 52.4 e | 5.5 c | 22.3 k | 18.2 m | 8.4 e |
| CC27Vu | 166.4 b | 9219.2 g | 677.0 g | 2079.0 d | 4904.9 b | 1483.1 h | 51.7 e | 7.5 b | 31.2 f | 23.1 g | 7.5 f |
| UI31Vu | 163.8 b | 9509.3 f | 1062.2 a | 2340.3 a | 3301.8 i | 2112.4 b | 54.9 d | 8.7 a | 36.7 b | 33.2 b | 9.9 d |
| BI30Vu | 156.4 b | 8865.6 h | 835.9 d | 2126.3 c | 3708.5 g | 1794.3 f | 42.8 g | 6.4 b | 22.0 k | 23.7 f | 7.5 f |
| BE29Vu | 155.8 b | 10193.1 c | 849.5 d | 1938.7 f | 4084.8 e | 1926.5 d | 52.4 e | 6.8 b | 38.5 a | 28.4 d | 8.7 e |
| CV47Vu | 140.3 c | 10398.3 b | 749.9 e | 1746.0 g | 3357.7 i | 1902.4 d | 56.4 c | 4.1 d | 29.3 h | 18.4 m | 7.6 f |
| SO12Vu | 127.9 c | 9157.6 g | 602.7 h | 1892.2 f | 3558.5 h | 1963.3 d | 40.5 h | 5.9 c | 34.5 d | 25.6 e | 12.8 b |
| MA41Vu | 109.6 c | 10656.3 a | 720.2 f | 2088.4 d | 4533.4 d | 2052.8 c | 42.2 g | 5.3 c | 28.4 i | 20.7 k | 14.3 a |
| Mean ± stDev^c^ | 179.9±42.8 | 9808.2±542.1 | 823.0±114.8 | 2045.0±140.4 | 4151.9±587.8 | 1919.6±175.5 | 52.3±8.7 | 6.2±1.3 | 30.8±4.7 | 23.6±5.6 | 9.6±2.0 |
| NRVs ^d^ (mg) |  | 2000 | 800 | 375 | 700 |  | 14 | 1 | 10 | 2 |  |

^a^ Average values of three separate determinations (n=3); ^b^ Homogeneous groups: accessions sharing the same letter for each mineral are not statistically diﬀerent according to the Scott-Knott test at 5% of confidence;
^c^ Standard deviation; ^d^ Nutrient reference values for daily reference intakes for minerals (Adults) [64].

**Table S5.** Genome size of the 33 *Phaseolus vulgaris* and *Vigna unguiculata* accessions estimated by flow cytometry.

| **Accession** | **Genome size (Mbp)** | | **H.G.**^b^ |
| --- | --- | --- | --- |
|  | **Average** | **stDev** ^a^ |  |
| BE29Vu | 1598.5 | 5.4 | a |
| BI30Vu | 1543.4 | 16.6 | b |
| MP34Vu | 1459.3 | 19.4 | c |
| SO12Vu | 1435.5 | 11.7 | c |
| LU33Vu | 1416.6 | 21.5 | d |
| HI25Vu | 1400.5 | 7.2 | d |
| MA24Vu | 1391.8 | 21.8 | d |
| MO28Vu | 1387.1 | 22.3 | d |
| CU17Pv | 1384.1 | 10.3 | d |
| CU15Pv | 1383.4 | 11.2 | d |
| CN32Vu | 1381.4 | 11.1 | d |
| SO14Pv | 1375.0 | 3.9 | e |
| SO8Pv | 1365.3 | 3.2 | e |
| MA21Pv | 1364.4 | 18.2 | e |
| CV36Vu | 1360.9 | 16.2 | e |
| CV38Pv | 1360.6 | 13.0 | e |
| MA41Vu | 1356.2 | 7.2 | e |
| CU18Pv | 1350.5 | 3.5 | e |
| HI19Pv | 1342.8 | 4.2 | f |
| SO9Pv | 1341.0 | 10.7 | f |
| SO10Pv | 1340.3 | 11.5 | f |
| CC27Vu | 1335.2 | 9.0 | f |
| MP02Pv | 1328.4 | 6.8 | f |
| MP04Vu | 1321.6 | 31.9 | f |
| MA43Pv | 1320.9 | 38.8 | f |
| MA42Pv | 1319.1 | 23.3 | f |
| CU16Pv | 1317.3 | 10.5 | f |
| SO11Pv | 1316.6 | 21.6 | f |
| CV49Pv | 1312.9 | 6.6 | f |
| SO7Pv | 1301.2 | 14.8 | g |
| SO13Pv | 1294.0 | 5.9 | g |
| MA20Pv | 1284.1 | 12.6 | g |
| CV47Vu | 1277.3 | 11.2 | g |

^a^ Standard deviation; ^b^ Homogeneous groups—accessions sharing the same letter are not statistically different according to the Scott-Knott test at 5% of confidence.

**Table S6**. Comparison of the average genome size of the *Vigna unguiculata* accessions estimated by flow cytometry groups by country of origin.

| **Country** | **Genome size (Mbp)** | | **H.G.^b^** |
| --- | --- | --- | --- |
|  | **Average** | **stDev ^a^** |  |
| Angola | 1429.4 | 88.0 | a |
| Mozambique | 1405.5 | 67.2 | a |
| Cabo Verde | 1360.9 | 16.2 | a |

^a^ Standard deviation; ^b^ Homogeneous groups: accessions sharing the same letter for each mineral are not statistically diﬀerent according to the Scott-Knott test at 5% of confidence.

**Table S7**. Comparison of the average genome size of the *Phaseolus vulgaris* accessions estimated by flow cytometry groups by country of origin.

| **Country** | **Genome size (Mbp)** | | **H.G.^b^** |
| --- | --- | --- | --- |
|  | **Average** | **stDev ^a^** |  |
| Angola | 1337.6 | 36.5 | a |
| Mozambique | 1335.6 | 30.7 | a |
| Cabo Verde | 1324.9 | 36.9 | a |

^a^ Standard deviation; ^b^ Homogeneous groups: accessions sharing the same letter for each mineral are not statistically diﬀerent according to the Scott-Knott test at 5% of confidence.

**Table S8**. Comparison of the average genome size of the *Vigna unguiculata* accessions estimated by flow cytometry groups by province/island of origin.

| **Province and country** | **Genome size (Mbp)** | | **H.G.^b^** |
| --- | --- | --- | --- |
|  | **Average** | **stDev ^a^** |  |
| Benguela, Angola | 1598.5 | 5.4 | a |
| Bié, Angola | 1543.4 | 16.6 | b |
| Sofala, Mozambique | 1435.5 | 11.7 | c |
| Luanda, Angola | 1416.6 | 21.5 | c |
| Huíla, Angola | 1400.5 | 7.2 | c |
| Maputo, Mozambique | 1390.4 | 73.7 | c |
| Moxico, Angola | 1387.1 | 22.3 | c |
| Cuanza Norte, Angola | 1381.4 | 11.1 | c |
| Malanje, Angola | 1374.0 | 24.1 | c |
| Santiago, Cabo Verde | 1360.9 | 16.2 | c |
| Cuando Cubango, Angola | 1335.2 | 9.0 | c |

^a^ Standard deviation; ^b^ Homogeneous groups: accessions sharing the same letter for each mineral are not statistically diﬀerent according to the Scott-Knott test at 5% of confidence.

**Table S9.** Comparison of the average genome size of the *Phaseolus vulgaris* accessions estimated by flow cytometry groups by province/island of origin.

| **Province and country** | **Genome size (Mbp)** | | **H.G.^b^** |
| --- | --- | --- | --- |
|  | **Average** | **stDev ^a^** |  |
| Cunene, Angola | 1356.7 | 30.9 | a |
| Huíla, Angola | 1342.8 | 4.2 | a |
| Sofala, Mozambique | 1336.4 | 32.2 | a |
| Maputo, Mozambique | 1328.4 | 6.8 | b |
| Santiago, Cabo Verde | 1324.9 | 36.9 | b |
| Malanje, Angola | 1317.0 | 36.4 | b |

^a^ Standard deviation; ^b^ Homogeneous groups: accessions sharing the same letter for each mineral are not statistically diﬀerent according to the Scott-Knott test at 5% of confidence.
